# Multiple Classes of Immune-Related Proteases Associated with the Cell Death Response in Pepper Plants

**DOI:** 10.1371/journal.pone.0063533

**Published:** 2013-05-16

**Authors:** Chungyun Bae, Su-min Kim, Dong Ju Lee, Doil Choi

**Affiliations:** 1 Department of Plant Sciences, Plant Genomics and Breeding Institute, Seoul National University, Seoul, Korea; 2 Higher Education Center for Bioregulator Research, Chonnam National University, Gwangju, Korea; Ghent University, Belgium

## Abstract

Proteases regulate a large number of biological processes in plants, such as metabolism, physiology, growth, and defense. In this study, we carried out virus-induced gene silencing assays with pepper cDNA clones to elucidate the biological roles of protease superfamilies. A total of 153 representative protease genes from pepper cDNA were selected and cloned into a *Tobacco rattle virus*-ligation independent cloning vector in a loss-of-function study. Silencing of 61 proteases resulted in altered phenotypes, such as the inhibition of shoot growth, abnormal leaf shape, leaf color change, and lethality. Furthermore, the silencing experiments revealed that multiple proteases play a role in cell death and immune response against avirulent and virulent pathogens. Among these 153 proteases, 34 modulated the hypersensitive cell death response caused by infection with an avirulent pathogen, and 16 proteases affected disease symptom development caused by a virulent pathogen. Specifically, we provide experimental evidence for the roles of multiple protease genes in plant development and immune defense following pathogen infection. With these results, we created a broad sketch of each protease function. This information will provide basic information for further understanding the roles of the protease superfamily in plant growth, development, and defense.

## Introduction

Proteases catalyze the hydrolytic cleavage of peptide bonds, which are present in all living organisms and play crucial roles in many biological processes [1]. Higher plants are autotrophic organisms that can synthesize all of their organic molecular components from inorganic nutrients without digestion of heterotrophic protein. However, hundreds of proteases are encoded by the plant genome, suggesting that these enzymes have essential roles in various plant processes including responses to developmental and environmental cues, metabolism, and immunity [2]–[4].

Proteases are classified into five families according to their catalytic activity, namely the cysteine, serine, threonine, metallo- and aspartic proteases based on their nucleophile and oxyanion stabilizer [5]. Depending on the catalytic mechanism, serine, cysteine, and threonine proteases use a portion of their amino acids as the catalytic site for the nucleophile, whereas metallo- and aspartic proteases use an activated water molecule as the nucleophile [6].

Several members of the serine, cysteine, and threonine protease families have reported regulatory roles in the development and morphogenesis of different stages of the plant life cycle. For instance, defective kernel 1 (DEK1), a cysteine protease, is involved in an epidermal cell fate stage, and a *dek1* mutant in maize (*Zea mays*) causes a lethal defect in which kernels lack an aleurone layer [7], [8]. Two other cysteine proteases, ubiquitin C-terminal hydrolase1 (UCH1) and 2 (UCH2), regulate branching in *Arabidopsis thaliana*. The *uch1/uch2* mutant strain exhibits reduced branched primary inflorescence under short day conditions [9]. In addition, the serine protease abnormal leaf shape 1 (ALE1) function in embryo cuticle deposition as evidenced by the lack of cuticle in embryos of *ale1* mutants [10].

Along with these roles in development, many aspartic and metalloproteases have been associated with immune defense in plants. Constitutive disease resistance 1 (*CDR1*) from *Arabidopsis,* an aspartic protease localized in the apoplast of plant cells, induces local and systemic defense responses. Overexpression of *CDR1* caused dwarfing and resistance to the virulent pathogen *Pseudomonas syringae* and *CDR1* deficiency resulted in increased susceptibility to infection by this pathogen [2], [11]. Aspartic proteases (APs) from *Solanum tuberosum* exhibit antimicrobial activity induced by abiotic and biotic stress by interacting directly with microbial cell surface proteins followed by membrane permeabilization [12]. Furthermore, APs from barley, which are specifically expressed in nuclear cells during degeneration, is reported to be involved in programmed cell death [13]. Moreover, matrix metalloproteases 2 isolated from *Glycine max* is induced by biotic stresses such as fungal or bacterial pathogens [14]. Altogether these reports suggest that proteases may play roles in the immune response against pathogenic infection in plants.

Functional characterization of plant protease gene family members has been conducted in a fragmentary scale to uncover a specific function for each protease. To understand the broad role of proteases in plants, we isolated the protease superfamily from pepper (*Capsicum annuum*) expressed sequenced tags (ESTs, http://genepool.kribb.re.kr/pepper/) on the basis of MEROPS (http://merops.sanger.ac.uk/) classification [15], [16]. We analyzed multiple pepper ESTs to gain a better understanding of proteases involved in plant development and immune defense. In an effort to identify protease function, a *Tobacco rattle virus* (TRV)-based virus-induced gene silencing (VIGS) technique was adapted as an easy and powerful method for silencing uncharacterized genes on a large scale [17]. To date, VIGS has been used successfully in studies of *Nicotiana benthamiana*, *Solanum lycopersicum*, *Solanum bulbocastanum*, *C. annuum*, *Arabidopsis,* and *Papaver somniferum* among other plants [18]–[23].

In this study, we present the phenotypic profile of *N. benthamiana* plants following silencing of multiple proteases. We further characterized some selected proteases with roles in pathogen-induced plant cell death. This study of 153 different protease genes from pepper plant could provide an overall sketch of the biological roles of plant proteases in growth, development, and defense against pathogens.

## Materials and Methods

### Plant Growth and Agroinfiltration

*N. benthamiana* plants were grown in a 4.09 inch soil pot. The pots were grown in a growth chamber at 22°C under a cycle of 16 h light and 8 h dark. TRV-based VIGS on *N. benthamiana* was performed as described by Dong et al. [24]. The phenotypic changes of protease-silenced plants were observed at 4 or 5 weeks after infiltration.

### Dataset

The database searched for annotated proteases was pepper EST database (http://genepool.kribb.re.kr/pepper/) [15]. Functional annotation for protease domain prediction was performed using Hmmpfam and Hmmsmart with E-value set at less than e-5. The annotated proteases were classified based on MEROPS database classification (http://merops.sanger.ac.uk/) [16]. In addition, *Arabidopsis, Populus trichocarpa* proteases gene family database and *N. benthamiana*, *S. lycopersicum*, *Solanum phureja* genome database were used for ortholog search [25], [26], [3], [27], [28].

### Cloning of Pepper EST for VIGS

Pepper ESTs were amplified with: 5′-GACGACAAGACCCT (adaptor sequence) - GTAATACGACTCACTATAGGGC (pBluescript SK- specific sequence: ESTs were packaged into pBluescript SK-; Stratagene) - 3′ and 5′-GAGGAGAAGAGCCCT (adaptor sequence) - CGCTCTAGAACTAGTGGATCC (pBluescript SK- specific sequence)-3′. The PCR products were purified with DNA Clean and Concentrator™ (Zymo Research) to remove primers and nonspecific PCR products. A total 15 fmol of purified PCR product was treated with T4 DNA polymerase (Novagen) in 10×reaction buffer containing 5 mM dATP at 22°C for 30 min and 70°C for 20 min for inactivation of T4 DNA polymerase. The TRV-LIC vector was digested with *Pst*I and treated with T4 DNA polymerase similarly with dTTP instead of dATP [24]. A total 15 fmol of T4 DNA polymerase treated PCR products and TRV-LIC vector were mixed and incubated at 65°C for 1 min and slowly cooled down to room temperature. Then, the mixture was transformed into *E. coli* DH5α competent cells. Transformants were tested by PCR amplification using primers 5′-TGTTACTCAAGGAAGCACGATGAGCT- 3′ and 5′- CAGGCACGGATCTACTTAAAGAACGTAG- 3′.PCR products with EST insertions were confirmed by DNA sequencing (NICEM, http://nicem.snu.ac.kr/).

### RNA Extraction and qRT-PCR Analysis

Total RNA was extracted from leaves of *N. benthamiana* plant using TRI Reagent (Molecular Research Center) according to the manufacturer’s instruction. First-strand cDNA was synthesized using 5 µg of total RNA in a mixture with anchor primer (oligo-dT) and SUPERSCRIPT® II Reverse-Transcriptase (Invitrogen) according to the manufacturer’s protocol. RT-PCR products were used for quantitative RT-PCR to monitor gene expression levels in protease-silenced plants. Triplicate samples from 3 independently silenced plants of cDNA were analyzed using Rotor-Gene 6000 apparatus (QIAGEN) with SYBR Green (Invitrogen) according to manufacturer’s instructions. All calculations and statistical analyses were demonstrated as described by the manufacturer. Primers for qRT-PCR of specific proteases were presented in Table S3. To normalize the expression levels, transcript level of *NbActin* gene was used as a control.

### Pathogen Preparation and Inoculation

The bacterial strains used for this study were *P. syringae* pv. *tomato* T1 which causes hypersensitive response (HR) cell death and *P. syringae* pv. *tabaci* which causes wild fire disease on *N. benthamiana* [29], [30]. Both bacterial pathogens were grown in yeast extract peptone (YEP) medium and resuspended in 10 mM sterile MgCl_2_ solution. *N. benthamiana* leaves were pressure infiltrated using a needless syringe with bacterial concentration of OD_600_ = 0.2 (*P. syringae* pv. *tomato* T1) and OD_600_ = 0.005 (*P. syringae* pv. *tabaci*). Changes of the HR cell death and symptom development in bacteria-infiltrated plants were observed for 3 and 7 days, respectively [31].

*P. syringae* pv. *tabaci* cultures (OD_600_ = 0.005, approx. 1×10^8^ CFU ml^−1^) were resuspended in 10 mM MgCl_2_ and infiltrated into the protease-silenced leaves using a 1 ml needless syringe. The growth of bacterial in the leaf was measured on four replicate plants. Two leaf discs (1 cm in diameter) per plant were collected from the *P. syringae* pv. *tabaci* infiltrated region of each protease-silenced and control plants. Numbers of the bacteria were measured by grinding the leaf discs in 10 mM MgCl_2_ and plating serial dilutions LB agar plants containing 100 ug ml^−1^ rifampicin on a daily basis for 2 dpi.

### Staining with Trypan Blue

To detect the cell death, leaves were stained with lactophenol/trypan blue (10 ml glycerol, 10 ml lactic acid, 10 g phenol and 10 mg trypan blue dissolved in 10 ml distilled water). Pathogen-infected leaves were vacuum-infiltrated two-times for 5 min in staining solution and incubated for overnight. Stained leaves were de-stained in 2.5 g ml^−1^ chloral hydrate (TOKYO KASEI). The de-stained leaves were mounted in 50% glycerol and photographed under a digital microscope (DIMIS M, Siwon Optical Technology, http://www.dimis.co.kr/) [32].

### Measurement of Ion Leakage

One and two days after infiltration with *P. syringae* pv. *tomato* T1, two leaf discs (1 cm in diameter) were floated in 5 ml of distilled water for 4 h at room temperature. Electrical conductivity was measured using a conductivity meter (EC215, HANNA instruments).

## Results

### Isolation of Putative Protease Genes in Pepper ESTs

The pepper EST database, which is a useful collection for functional genomics, consists of 122,582 ESTs including 22,011 unigenes, 11,225 consensus sequences and 5,585 full-length cDNAs have been published [15]. From the pepper EST database, putative protease genes (totaling 939 entries, data not shown) were selected computationally using Hmmpfam and Hmmsmart for protease domain prediction based on classifications from MEROPS. This protease database, displays hierarchical classifications and homologous proteases grouped into species, families, and clans based on evolutionary relationships. MEROPS can also be used to obtain information about the substrate and inhibitors of particular protease [16].

To select representative protease genes, sequence alignments using TBLASTX, BLASTX, and BLASTP were performed to avoid silencing multiple genes with homology. Ultimately, 153 pepper cDNAs were selected as representatives of protease genes for VIGS. The 153 pepper cDNA is presented with its EST ID, MEROPS ID, e-value, and the accession numbers of corresponding from *N. benthamiana*, *S. lycopersicum*, *S. phureja*, *Arabidopsis* genome database and other organisms are summarized in Table S1 [25]–[28]. The selected protease genes consisted of 15 aspartic, 27 cysteine, 28 metalloproteases, 72 serine, and 11 threonine proteases as classified by MEROPS. All of the selected cDNAs were cloned into the TRV-LIC vector for the VIGS assay [24].

### Phenotypic Analysis of Protease-silenced Plants

VIGS experiments were performed in *N. benthamiana* plants with the selected 153 cDNAs. Since pepper and *N. benthamiana* belong to the same solanaceae family, coding sequences from both plants exhibit a high degree of similarity. Previous studies have also demonstrated successful heterologous silencing of tomato genes in *N. benthamiana* [24]. Therefore, it is not surprising that several genes from pepper have been characterized in *N. benthamiana* using the VIGS assay [33], [30], [32]. The pepper ESTs investigated in this study are listed with their corresponding *N. benthamiana* proteases in Table 1.

**Table 1:** List of pepper EST referred in this study with its corresponding proteases in *N. benthamiana.*

		*N. benthamiana* genome	
Pepper EST ID^a^	Classification^b^	Accession number^c^	Description
Ncn10583	M18	NbS00031969g0005.1	Aspartyl aminopeptidase protein
Ncn1321	T01	NbS00028089g0021.1	Proteasome subunit alpha
Ncn1998	S33	NbS00039951g0005.1	Proline iminopeptidase
Ncn2132	M24	NbS00024034g0008.1	Xaa Pro dipeptidase
Ncn2134	T01	NbS00053635g0009.1	Proteasome subunit beta type
Ncn2155	C15	NbS00016770g0011.1	Pyrrolidone carboxylate peptidase
Ncn2258	A01	NbS00031482g0006.1	Aspartic proteinase
Ncn2901	A01	NbS00004524g0008.1	Aspartic proteinase nepenthesin 1
Ncn3606	S33	NbS00008723g0009.1	Hydrolase alpha/beta fold
Ncn4597	S10	NbS00017915g0109.1	Niben044Scf00017915∶116939.122486
Ncn4707	M16	NbS00026030g0004.1	Peptidase M16 family
Ncn5036	S01	NbS00055986g0001.1	Serine protease
Ncn5964	A01	NbS00059099g0001.1	Aspartic proteinase nepenthesin 1
Ncn6721	M24	NbS00011972g0104.1	Niben044Scf00011972∶154949.175101
Ncn7004	C26	NbS00007507g0016.1	Glutamine amidotransferase
Ncn7288	T01	NbS00043198g0009.1	Proteasome subunit alpha type
Ncn8326	M18	NbS00026037g0013.1	Aspartyl aminopeptidase
Ncn881	M24	NbS00006055g0014.1	Proliferation associated protein 2G4
Ncn8849	T01	NbS00021500g0008.1	Proteasome subunit alpha type
Ncn9390	A01	NbS00031201g0007.1	Nucellin aspartic protease fragment
Ncn964	S01	NbS00023513g0009.1	Serine protease Do
Ncn9826	M24	NbS00009510g0004.1	Xaa pro aminopeptidase
N n7292	C19	NbS00037871g0005.1	Ubiquitin carboxyl terminal hydrolase

a Pepper (*C. annuum*) EST database (http://genepool.kribb.re.kr/pepper/).

b Based on MEROPS database (http://merops.sanger.ac.uk/) classification.

c *N. benthamiana* genome database (http://solgenomics.net/, Niben.genome.v0.4.4).

**Figure 1:**
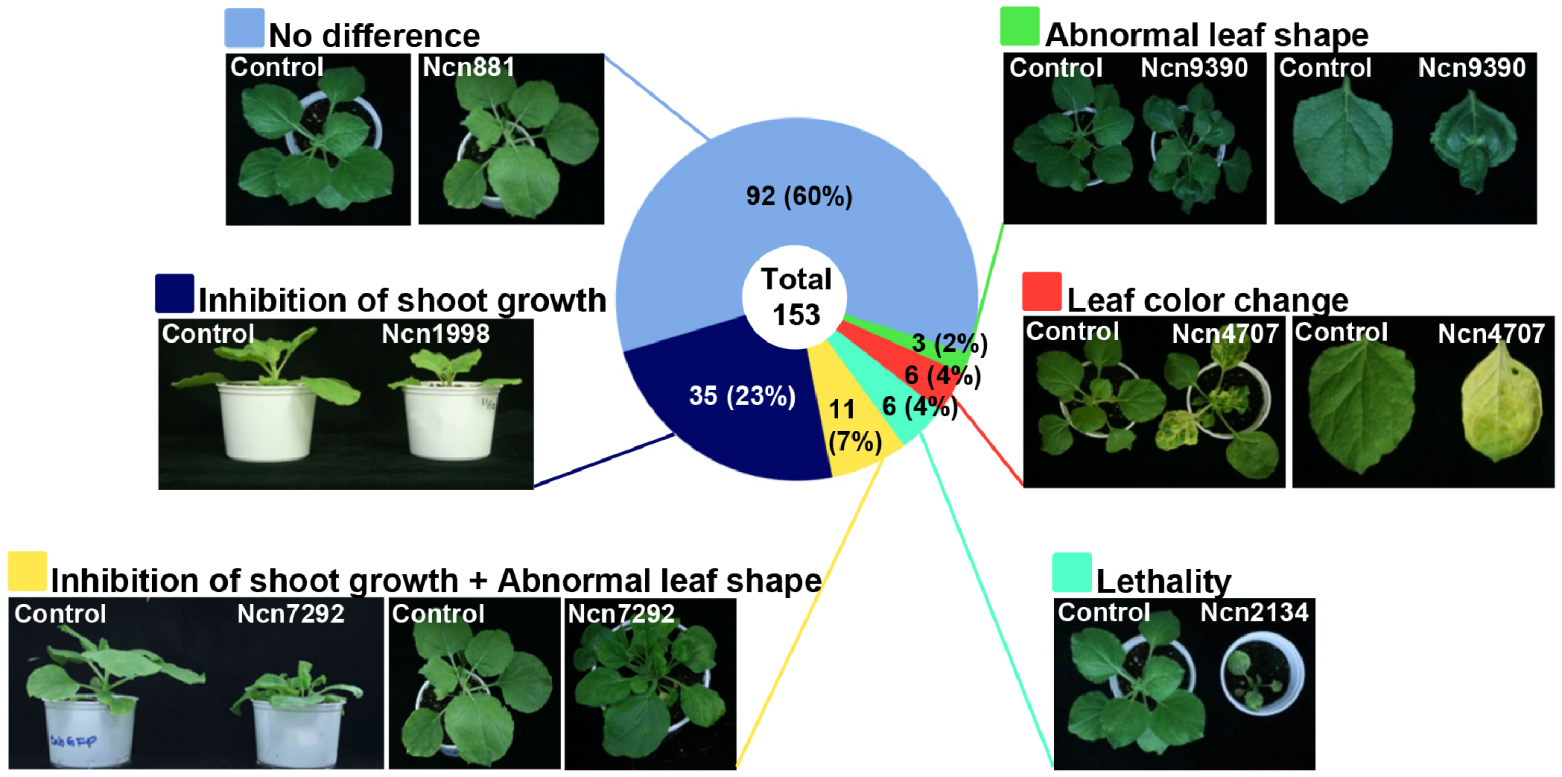
Phenotypic classification of protease-silenced plants. Protease-silenced plants are categorized into 6 classes. Each class was given with a specific color square box on left side of the class description. Circular diagram indicates the percentage each class. Representative protease described in each class is shown. These phenotype changes had been observed for 3 or 4 weeks and the picture are taken at 3 or 4 weeks after silencing. For every protease gene, 4 plants were silenced at each experiment. Similar results were obtained from at least three independent experiments. One representative experiment is shown.

The developmental phenotypes of protease-silenced plants were observed 3 or 4 weeks after VIGS and representative examples were shown (Fig. 1 and Table S2). These phenotypes were grouped into six classes, including no difference, inhibition of shoot growth, inhibition of shoot growth with abnormal leaf shape, lethality, leaf color change, and abnormal leaf shape (Fig. 1). The largest class, of which *proliferation-association protein 1* silencing is an example *(Ncn881*, Fig. 1), showed no phenotypic differences compared to the control plant and was composed of 92 proteases. The second largest class contained 35 proteases and gene silencing resulted in severe growth retardation relative to control plants (Fig. S1A), shown in the silencing phenotype of *prolyl aminopeptidase* (*Ncn1998*, Fig. 1). The third largest class consisted of 11 proteases and the silencing phenotype was characterized by severe stunting with crinkled leaves (Fig. S1B). This is evidenced by an ortholog of *At5g22030* (*Arabidopsis ubiquitin-specific protease 8*) (*Ncn7292*, Fig. 1). The fourth largest class, composed with 6 proteases (Fig. S1C), was characterized by lethality as shown by the silencing phenotype of *proteasome subunit beta 3* (*Ncn2134*, Fig. 1). The fifth class contained 6 proteases (Fig. S1D) and silencing resulted in photobleaching and yellowing of leaves as shown by *chloroplast* (*stromal*) *processing peptidase* silencing *(Ncn4707*, Fig. 1). This altered phenotype showed similar developmental phenotypes to those in previous studies of down-regulation of the gene product [34], [35]. The smallest class was made up of 3 proteases (Fig. S1E) that, when silenced, lead to disruption of leaf development resulting crinkled leaves as shown by silencing an ortholog of *At1g49050 (aspartyl protease family) protein-type peptidase* (*Ncn9390*, Fig. 1).

Nearly half of the members exhibited altered phenotypes in aspartic, cysteine and metalloproteases family (aspartic: 9 out of 15, cysteine: 14 out of 27, metallo-: 13 out of 28) (Table S2). However, the serine protease family was the least affected group with only 16 out of 72 members displaying a phenotypic change compared to that of control plants.

Interestingly, 9 out of 11 threonine protease family members produced altered developmental phenotypes, such as severe stunting, crinkled leaves, and lethality. Consistant with these results, previous studies have demonstrated that threonine proteases play a crucial role in the 26S proteasome and regulation of all aspects of plant development [36], [37]. These data suggest that threonine proteases have an essential role in plant growth and development.

### Temporal Change in Hypersensitive Response (HR) Cell Death in Protease-silenced Plants

**Figure 2:**
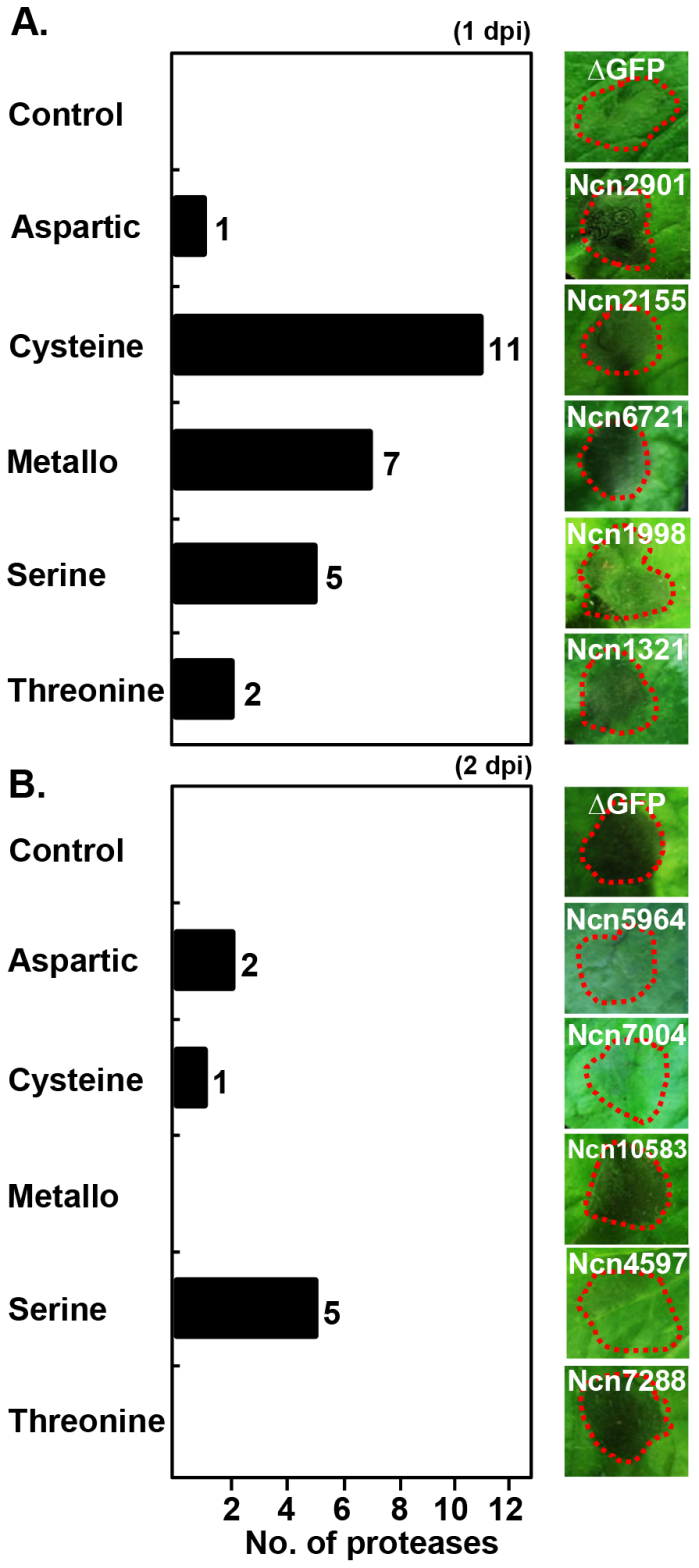
Change of HR cell death in protease-silenced plant following avirulent bacterial pathogen inoculation. Protease-silenced plants were infiltrated with avirulent bacterial pathogen *P. syringae* pv. *tomato* T1 (OD_600_ = 0.2). The HR cell death symptoms were taken at 1 dpi (A) and 2 dpi (B). The bar graph indicates the numbers of showing enhanced (A) or delayed (B) HR cell death from each protease family (left panel). The right panel shows the representative member from each family representing enhanced (A) and delayed (B) HR cell death. For every protease gene, 6 sections per 1 leaf were infected with the pathogen and total 2 leaves were used for 1 plant. Total 4 plants were infiltrated at each experiment. Similar results were obtained from at least three independent experiments. Red dotted line indicates the site of *P. syringae* pv. *tomato* T1 infection. One representative experiment is shown.

To investigate the role of proteases in immune defense, an avirulent pathogen *P. syringae* pv.*tomato* T1 capable of inducing HR cell death in *N. bethamiana* was introduced into protease-silenced plants [38]. Three weeks after silencing, plants were inoculated with a suspension of *P. syringae* pv. *tomato* T1 at a density of OD_600_ = 0.2. HR cell death was observed at days 1 and 2 post-inoculation (1 dpi and 2 dpi, respectively) in TRV-Δ*GFP* control plants. After 1 dpi, 26 protease-silenced plants showed temporally advanced HR cell death compared to that of control plants while the control plant showed HR cell death at 2 dpi. This group consisted of 11 cysteine, 7 metallo-, 5 serine, 2 threonine, and 1 aspartic protease family members (Fig. 2A and Fig. S2A). Cysteine protease deficiency produced the greatest effect on HR cell death in response to avirulent pathogen. This result is consistent with previous reports that demonstrated a crucial role for cysteine proteases in the plant-pathogen/pest interaction [39], [40]. After 2 dpi, delayed HR cell death was observed in 5 serine, 2 aspartic, and 1 cysteine protease-silenced plants. Delay of the HR cell death was until 3 dpi. Interestingly, none of the metallo- and threonine protease family members exhibited delayed HR cell death in gene-silenced *N. benthamiana* plants (Fig. 2B and Fig. S2B).

### Change of Disease Symptom Development in Protease-silenced Plants

**Figure 3:**
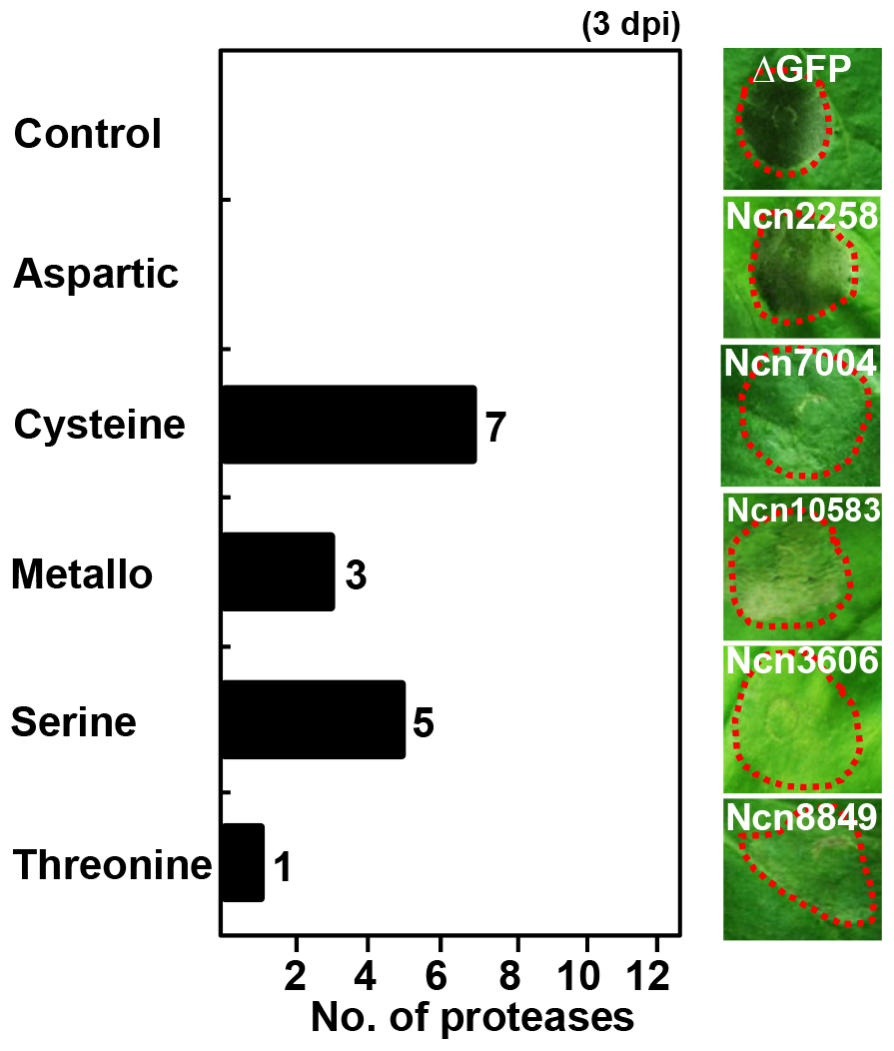
Change of disease symptom development in protease-silenced plant following virulent bacterial pathogen inoculation. Protease-silenced plants were infiltrated with virulent bacterial pathogen *P. syringae* pv. *tabaci* (OD_600_ = 0.005). The disease symptoms induced by the pathogen were taken at 3 dpi. The bar graph indicates the numbers of showing delayed disease development from each protease family (left panel). The right panel shows the representative member from each family which showed delayed symptom. For every protease gene, 6 sections per 1 leaf were infected with the pathogen and total 2 leaves were used for 1 plant. Total 4 plants were infiltrated at each experiment. Similar results were obtained from at least three independent experiments. Red dotted line indicates the site of *P. syringae* pv. *tabaci* infection. One representative experiment is shown.

To better understand the role of proteases in the immune response to pathogen invasion, protease-silenced *N. benthamiana* plants were also infected with a virulent bacterial strain. *P. syringae* pv. *tabaci,* a fire blight pathogen isolated from tobacco plant, produces a monocyclic ß-lactem named tabtoxin and causes loss of chlorophyll, accumulation of ammonia and finally inactivation of glutamine synthetase in plant [41], [42], [29]. Appearance of the susceptible host plant infected with *P. syringae tabaci* starts with water-soaking in the apoplast of the infected tissues, localized tissue necrosis and various tissue discolorations [29]. Protease-silenced and control plants were inoculated with *P. syringae* pv. *tabaci* at an OD_600_ = 0.005. Disease symptoms were observed at day 3 post-inoculation (3 dpi) in both groups. Sixteen protease-silenced plants constituting 7 cysteine, 5 serine, 3 metallo- and 1 threonine proteases showed delayed development of disease symptoms (Fig. 3 and Fig. S3). Disease symptom was delayed until 4 to 5 dpi. Aspartic proteases were not found in this experimental group.

### Characterization of Selected Protease-silenced Plants following Pathogens Infection

To confirm the level of gene silencing in each protease subfamily, quantitative reverse transcription-polymerase chain reaction (qRT-PCR) was conducted using primers that anneal outside the sequence used for silencing. Our data indicate that the level of mRNA transcript for all protease members was decreased at least 70% by gene silencing compared to control plants (Fig. S4).

Three protease subfamilies, which showed modulated pathogen responses when silenced, were selected for further characterization, including M18 (metallo- type, aminopeptidase family), M24 (metallo- type, methionyl aminopeptidase family) and S01 (serine type, chymotrypsin family). M18 family is consisted with a member showing delayed symptom development whereas M24 and S01 families are each constituted with a member resulted in enhanced and delayed HR cell death response compared to the control. Silencing of each representative member showed enhanced and delayed HR cell death and delayed disease symptom onset compared to the control, respectively.

**Figure 4:**
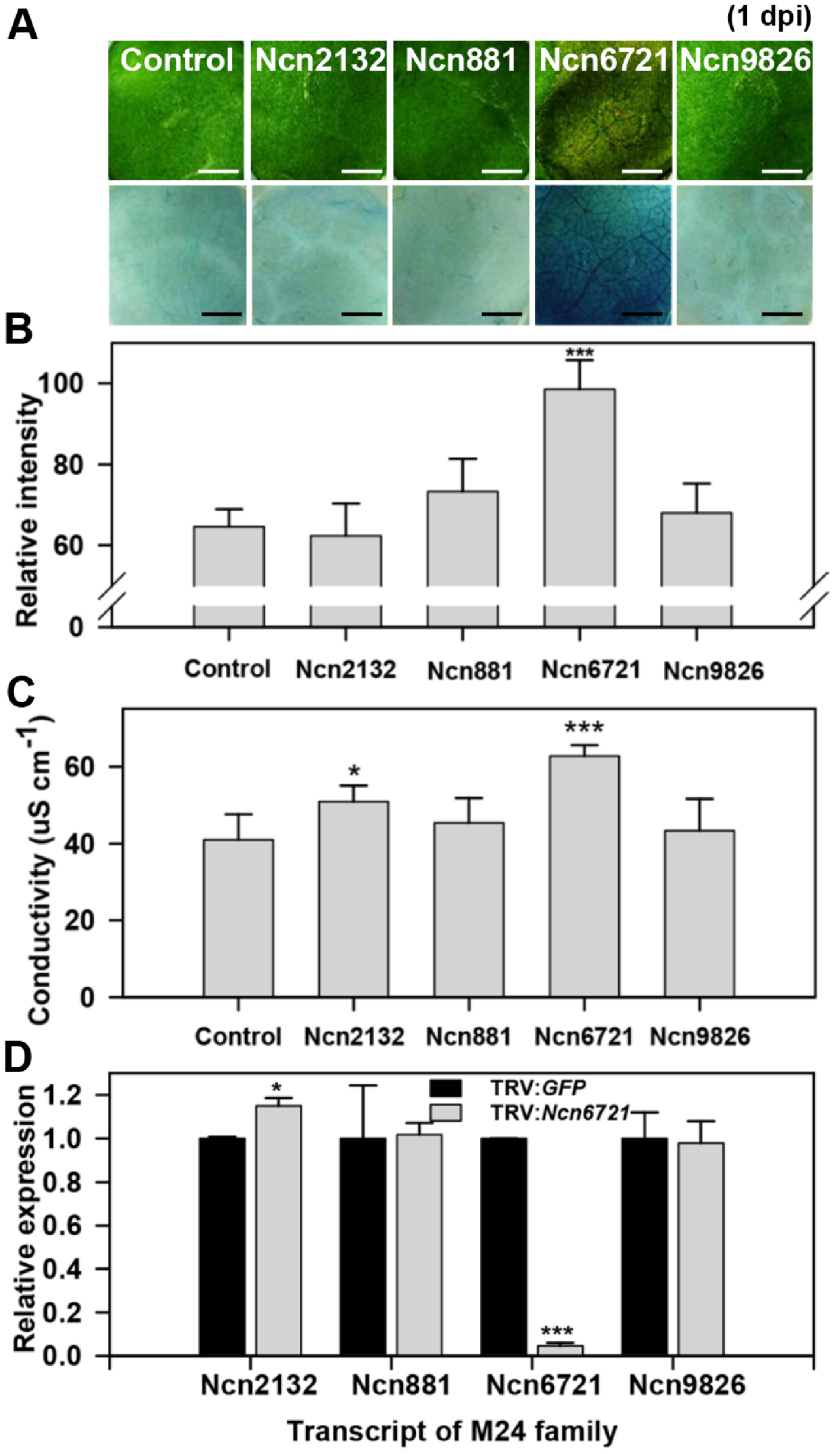
Enhanced HR cell death identified in M24 protease subfamily-silenced plant. A. HR cell death from M24 protease subfamily-silenced *N. benthamiana* plant following non-host pathogen infection (*P. syringae* pv. *tomato* T1, OD_600_ = 0.2). HR symptoms were taken at 1 dpi (upper panel). Trypan blue staining at 1 dpi on the same area as the photographs was taken (lower panel). Scale bars = 0.3 mm. B. Relative intensity of trypan blue stained *N. benthamiana* plants. Values are calculated as means and standard deviation (SD) for three plants (n = 3) of one infiltration experiment. Relative intensity was calculated to the control of Fig. 5A. C. Ion leakage from leaf discs of M24 protease subfamily-silenced *N. benthamiana* plant following non-host pathogen infection. Values are means±SD (n = 3). D. Transcript levels of M24 protease subfamily members were examined in *Ncn6721*-silenced plant. Values are means±SD (n = 3). The values were normalized to *NbActin* and were calculated to the control. Similar results were obtained from at least two experiments. One representative experiment is shown. Asterisks indicate significant differences relative to the control as determined by Student’s *t* test (*P<0.05, **P<0.01, ***P<0.001).

**Figure 5:**
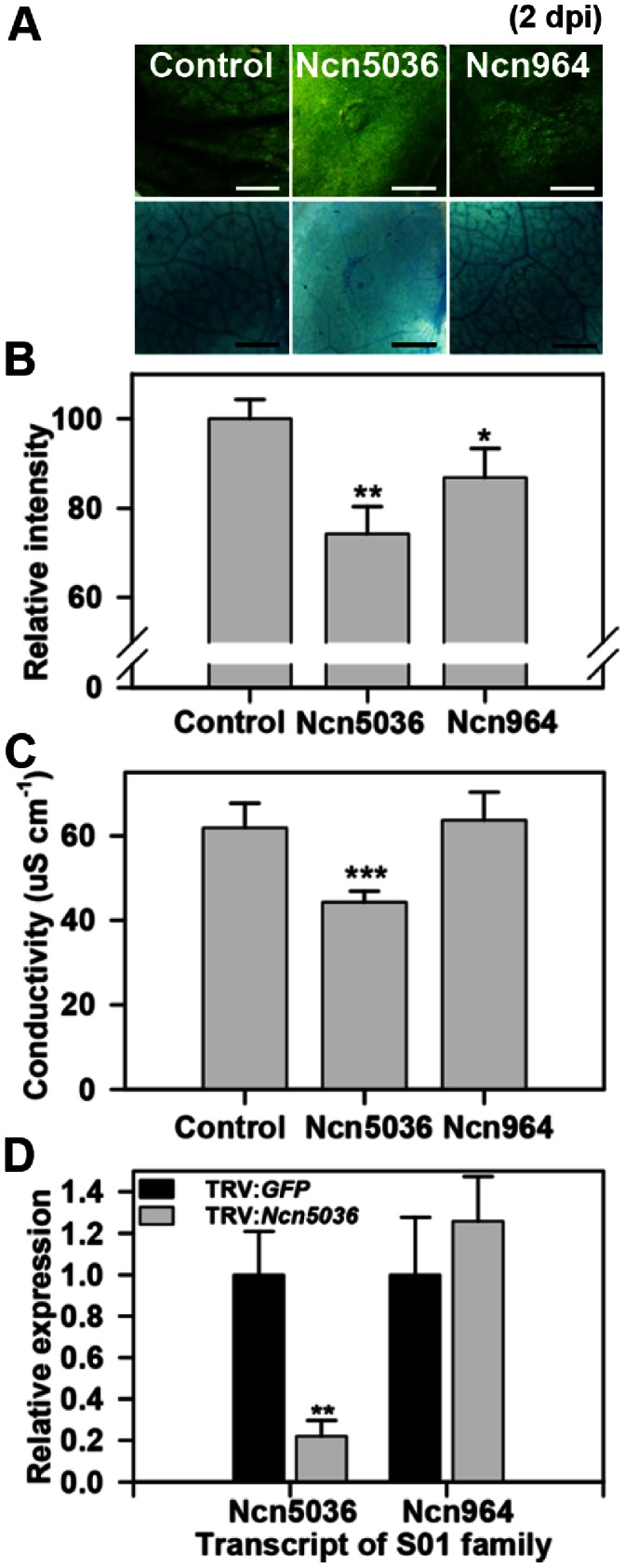
Delayed HR cell death obtained in S01 protease subfamily-silenced plant. A. HR cell death of S01 protease subfamily-silenced *N. benthamiana* plant following non-host pathogen infection (*P. syringae* pv. *tomato* T1, OD_600_ = 0.2). HR symptoms were taken at 2 dpi (upper panel). Trypan blue staining at 2 dpi on the same area as the photographs was taken (lower panel). Scale bars = 0.3 mm. B. Relative intensity of trypan blue stained *N. benthamiana* plants. Values are means±SD (n = 3). Relative intensity was calculated to the control. C. Ion leakage from leaf discs of S01 protease subfamily-silenced *N. benthamiana* plant following non-host pathogen infection. Values are means±SD (n = 3). D. Transcript level of S01 protease subfamily member was examined in *Ncn5036-* silenced plant. Values are means±SD (n = 3). The values were normalized to *NbActin* and were calculated to the control. Similar results were obtained from at least two experiments. One representative experiment is shown. Asterisks indicate significant differences relative to the control as determined by Student’s *t* test (*P<0.05, **P<0.01, ***P<0.001).

The M24 and S01 protease subfamilies possessed with a member showing enhanced and delayed HR cell death after infection with an avirulent pathogen, *P. syringae* pv. *tomato* T1, compared to control plants at 1 dpi and 2 dpi (upper panel of Fig. 4A and Fig. 5A). The control plants represented the HR cell death at 2 dpi, while the enhanced HR cell death of Ncn6721 was carried out at 1 dpi while the HR cell death of Ncn5036 was delayed until 3 dpi. Trypan blue staining was performed to assess cell death in the M24 and S01 protease subfamily member (lower panel of Fig. 4A and Fig. 5A). Our data reveal that cell death following *Ncn6721*-silencing was 20% higher than the control plants and other silenced member from the M24 protease subfamily at 1 dpi (Fig. 4B), suggesting a critical role for this gene product in plants. In contrast, cell death in *Ncn5036*-silenced plants was 20% lower than the control and *Ncn964*-silenced plants at 2 dpi (Fig. 5B). The ion leakage of the pathogen-infiltration area was significantly high in *Ncn6721*- and low in *Ncn5036*-silenced plant than that of the control plant (Fig. 4C and 5C).

Gene-specific silencing was confirmed with gene-specific primers in the *Ncn6721*-silenced plants. Among the closest paralogs, the transcript levels of *Ncn2132*, *Ncn881* and *Ncn9826* were not reduced in *Ncn6721*-silenced plants (Fig. 4D). Additionally, the level of *Ncn964* mRNA was not decreased in *Ncn5036*-silenced plants (Fig. 5D). The transcript levels of other M24 and S01 protease members in each protease-silenced plant are shown in Fig. S5. Interestingly, *Ncn6721* is predicted to encode methionyl aminopeptidase 2 (MAP2), a cytosolic enzyme responsible for protein N-terminal methionine excision (NME). This cytoplasmic NME machinery displays the same specificity in the plant and animal kingdom and has been established to be required for normal development [43]. Functional studies of MAP2 in plants during development were performed in *Arabidopsis* [44]. Our finding indicates that MAP2 may play a role in pathogen-induced HR cell death in plants. Moreover, *Ncn5036* is described as an At5g40200-type peptidase and predicted to encode degradation of periplasmic proteins 9 (DegP9), a serine type ATP-dependent protease. In *Arabidopsis,* DegP9 protease is involved in the degradation of D1 protein from photosystem II following photoinhibition and is up-regulated during senescence [45]–[47]. Several recent studies have confirmed that plant chloroplasts are associated with plant pathogen-induced cell death [48]. This result indicates that DegP9 is associated with HR cell death in plants.

**Figure 6:**
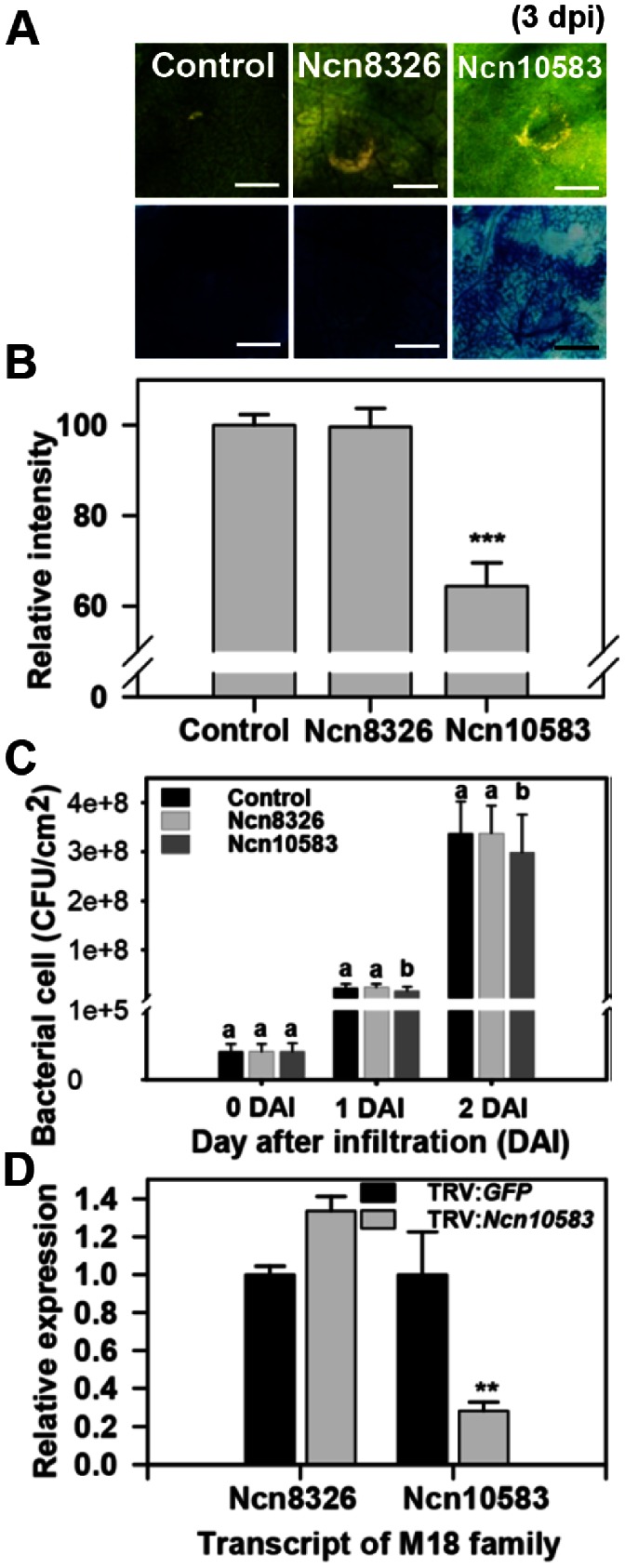
Delayed pathogen-induced disease symptom represented in M18 protease subfamily-silenced plant. A. Disease symptom of M18 protease subfamily-silenced *N. benthamiana* plant following bacterial host pathogen infection (*P. syringae* pv. *tabaci*, OD_600_ = 0.005). Disease symptoms were taken at 3 dpi (upper panel). Trypan blue staining is in the same area as the photographs were taken at 3 dpi (lower panel). Scale bars = 0.3 mm. B. Relative intensity of trypan blue stained *N. benthamiana* plants. Relative intensity was calculated to the control. Values are means±SD (n = 3). C. Bacterial cell growth in control and M19 protease subfamily-silenced *N. benthamiana* plants infiltrated with *P. syringae* pv. *tabaci* (1×10^8^ CFU ml^−1^ in MgCl_2_) was determined at 0, 1 and 2 dpi. Values are means±SD (n = 3). Different letters indicate significant differences at the 95% level by Duncan’s multiple range tests. D. Transcript level of M18 protease subfamily member was examined in *Ncn10583*- silenced plant. Values are means±SD (n = 3). The values were normalized to *NbActin* and were calculated to the control. Similar results were obtained from at least two experiments. One representative experiment is shown. Asterisks indicate significant differences relative to the control as determined by Student’s *t* test (*P<0.05, **P<0.01, ***P<0.001).

The M18 protease subfamily contains a member that is involved in delayed disease symptom development onset following infection with the virulent bacterial pathogen *P*. *syringae* pv. *tabaci* (Fig. 6). Disease symptom of *Ncn10583*-silenced plant was carried out at 4 to 5 dpi, whereas the control and *Ncn8326*-silenced plant showed disease symptom at 3 dpi (upper panel of Fig. 6A). Trypan blue analysis revealed that *Ncn10583*-silenced plants showed 30% less death than the control and *Ncn8326*-silenced plants at 3 dpi (lower panel of Fig. 6A and Fig. 6B). The bacterial cell growth of *Ncn10583*-silenced plants was one and a half-fold lower than in control-silenced plants (Fig. 6C). Gene-specific silencing of *Ncn10583* was also confirmed by qRT-PCR. The transcript level of the most closely related protease *Ncn8326* was not reduced in *Ncn10583*-silenced plants (Fig. 6D). The level of *Ncn10583* mRNA in *Ncn8326*-silenced plants is shown in Fig. S5. *Ncn10583* is an At5g60160-like peptidase and predicted to encode a Zn-dependent exopeptidase superfamily protein also known as aspartyl aminopeptidase (AAP), which is distributed widely in animals and plants and involved in a variety of physiologically important processes [49]. Most studies of AAP have been limited to mammals and focused on its functions and enzymatic activity [50], [51]. Nevertheless, our data imply that AAP plays a role as a susceptibility factor of plants against virulent bacterial pathogens.

## Discussion

This study presents a larger view of the role of protease in plants. Some of our results pertaining to the function of these enzymes were consistent with previous reports. Among the 61 proteases identified to be involved in the plant developmental process by loss-of function studies, 39 belong to the serine, cysteine, or threonine protease families. Several members from these families have been demonstrated to play roles in plant growth and development [7], [8], [10], [9].

Based on the EST reference dataset, our analysis of representative proteases provided further insight into the role of these enzymes in plant development. Our data demonstrate that silencing of *Ncn1998* (serine type) resulted in inhibition of shoot growth (Fig. 1, Fig. S1A, and Table S2). *Ncn1998* is predicted to encode a prolyl aminopeptidase (PAP) with a significant degree of sequence identity to other solanaceous PAP proteins in other species, namely *S. lycopersicum* PAP (88%, Solyc11g044310.1.1), *N. benthamiana* PAP (74%, NbS00039951g0005.1), *Medicago truncatula* PAP (92%, XP003593347.1), and *Vitis vinifera* PAP (84%, XP002271289.2). Despite the ubiquity of PAP in nature, its role remains poorly understood in plants. To date, studies have only shown that the mRNA level of *PAP* is increased in the shoots of triticale plants in response to suboptimal conditions such as drought and high saline [52]. Our results are the first to indicate that PAP may function in plant growth and development, including the control of shoot apical meristem formation. More specifically, proline, a product of PAP, has various physiological roles in plants, animals, and micro-organisms [53], [54]. Our results suggest that decreased expression of *PAP* may affect proline metabolism and cause an altered phenotype in *Ncn1998*-silenced plants. Our experiment was limited to observing the pathology of the silenced plant for only 3 or 4 weeks; therefore, either a longer observation period or examination of knock out mutants may needed to determine the role of *PAP* in reproductive growth processes such as floral development.

Multiple proteases have roles in the pathogenesis and immune defense of plants. Our study identified 34 proteases that function in avirulent pathogen-induced HR cell death; 7 metallo- and 12 cysteine family members represented these proteases. Moreover, we identified 16 proteases, of which 7 were members of the cysteine family, involved in the development of pathogen-induced disease symptoms. Many members of the aspartic, cysteine, and metalloprotease families have been reported as associated with plant defense responses [2], [14], [39], [40], [11]. Our results are in close agreement with previous reports. Interestingly, silencing of only 3 aspartic proteases affected HR cell death in response to infection by an avirulent pathogen, while aspartic proteases did not appear to play a role in disease symptom development in plants.

To expand our knowledge of the involvement of proteases in pathogen infection, one protease that showed delayed HR cell death and disease symptom development after silencing was selected for further study (Fig. 2B and Fig. 3). *Ncn7004*, an ortholog of At5g38200 in *Arabidopsis*, is predicted to encode a Class 1 glutamine amidotransferase (GAT1)-like protein and has significant similarity in other species, including *S. lycopersicum* (92%, Solyc02g086300.2.1), *V. vinifera* (93%, XP002279823.1), *N. benthamiana* (90%, NbS00007507g0016.1), and *Arabidopsis* (89%, NP564885.1).

GAT generates ammonia from glutamine amide nitrogen and transfers it to acceptors [55]. One bacterial pathogen we used to infect *GAT-1 like protein* (*Ncn7004*)-silenced plants was *P. syringae* pv. *tabaci*, which produces tabtoxin to inhibit the plant enzyme glutamine synthetase (GS) [56]. GS is a key enzyme in the photorespiratory nitrogen cycle. Inhibiting this enzyme in plants results in chlorosis, which arises due to necrotic lesions caused by bacteria on leaves [57]. Moreover, tabtoxin-induced symptoms are associated with the accumulation of ammonia under photorespiration [58]. Challenging *Ncn7004*-silenced plants with virulent bacterial pathogen resulted in delayed disease onset, possibly due to blocking glutamine amide nitrogen from supplying ammonia. This decreased level of ammonia may merely slow the development of disease since ammonia accumulation was not enough to cause the symptom. To date, studies on GAT have mainly focused on its biochemical and structural properties [55], [59]. Thus, our finding on the function of *GAT* may provide additional insight into the molecular basis of disease symptom development in tobacco plants.

In addition, the *Ncn7004*-silenced plant showed other phenotypes besides cell death and defense responses. The *Ncn7004*-silenced plant displayed inhibited shoot growth and abnormal leaf shape (Fig. S1B). Ncn7004 consists of a GAT type 1 domain and could function similar to what has been reported previously, such as the effect of lower nitrogen metabolism on normal plant growth [60]. Moreover, GAT links nitrogen metabolism to the biosynthetic pathways of several important compounds [55]. Nitrogen supply is also known to affect plant disease development [60]. In previous studies, GAT was reported clustered with *At1g15040*, which belongs to the same subfamily as GAT1 (GATase 1_2) in *Arabidopsis*. In the *Arabidopsis* genome, there are 30 putative genes encoding GAT1 and a previous study suggested that *At1g15040* has specific functions in plant branching control [61]. Our results also reveal that *Ncn7004* might have roles in plant developmental processes. Furthermore, HR cell death is a kind of programmed cell death (PCD), which is important throughout plant development stages such as senescence, vacuole formation for the degradation of cellular contents, normal seed development, leaf sculpting, cell death in the root cap, and shaping the sexual and nonsexual organs of the flower [62]. GAT1-like protein may play both processes, roles in plant developmental and pathogen-induced cell death processes, as PCD. Together with previous studies, our results suggest that *GAT1-like protein* (*Ncn7004)* is closely associated with plant development and the regulation of cell death in response to pathogen invasion in plant immunity.

A number of plant proteases have been implicated in HR development. Some of them were reported as have roles in regulating HR and some of them were firstly described in this study. Orchestration of the multiple proteases in HR cell death is not quite clear yet but each protease may has distinct and diverse roles. From previous studies, some proteases can act at the level of signal perception, transduction and execution in defense response [63]. There are some evidences that type I metacaspase is a initiator or activator of type II metacaspase for progression of PCD [64]. Moreover, *AtMC1* (*Arabidopsis metacaspase 1*) works as a positive regulator, whereas *AtMC2* function as a negative regulator of cell death [65]. Even with these studies, there are still more to be found in the events of the chain leading to cell death. Our data only have given the evidence for one-to-one interaction with a protease and a pathogen. Comprehensive studies on the functions of these proteases may be required to uncover the multiple roles of proteases in the complicated machinery of HR cell death in plants. Therefore, our data might have given evidence for the starting point of the cell death event as the diverse roles of protease in HR cell death.

In this study, we provide a phenotypic sketch of protease function in plant developmental processes, pathogen-induced disease symptom development, and HR cell death. Our studies, together with previously published data, provide additional insights into the unknown role of proteases and clues for further investigation into the molecular functions of the protease super family in plants.

## Supporting Information

Figure S1 **Altered phenotypes of protease-silenced plants.** Protease-silenced plants showing altered phenotypes are categorized into 5 classes. The phenotypes are A. Inhibition of shoot growth. B. Inhibition of shoot growth with abnormal leaf shape. C. Lethality. D. Leaf color change. E. Crinkled leaves. These phenotype changes had been observed for 3 or 4 weeks and the picture are taken at 3 or 4 weeks after silencing. For every protease gene, 4 plants were silenced at each experiment. Similar results were obtained from at least three independent experiments. One representative experiment is shown.
(TIF)

Figure S2 **Enhanced and delayed HR responses in protease silenced-plant following incompatible pathogen infection.** Protease-silenced plants were infiltrated with non-host bacterial pathogen *P. syringae* pv. *tomato* T1 (OD_600_ = 0.2). The HR cell death symptoms were taken at 1 dpi (A) and 2 dpi (B). The phenotypes indicate enhanced (A) or delayed HR (B) responses. For every protease gene, 6 sections per 1 leaf were infected with the pathogen and total 2 leaves were used for 1 plant. Total 4 plants were infiltrated at each experiment and done it at least three repeated tests. Red dotted line indicates the site of *P. syringae* pv. *tomato* T1 infection. One representative experiment is shown.
(TIF)

Figure S3 **Delayed symptom development in protease-silenced plant following compatible pathogen infection.** Protease-silenced plants were infiltrated with host bacterial pathogen *P. syringae* pv. *tabaci* (OD_600_ = 0.005). The disease symptoms induced by the pathogen were taken at 3 dpi. For every protease gene, 6 sections per 1 leaf were infected with the pathogen and total 2 leaves were used for 1 plant. Total 4 plants were infiltrated at each experiment and done it at least three repeated tests. Red dotted line indicates the site of *P. syringae* pv. *tabaci* infection. One representative experiment is shown.
(TIF)

Figure S4 **Confirmation of gene-specific silencing in M24, S01 and M18 proteases subfamily.** Silencing confirmation of M24 (A), S01 (B) and M18 (C) protease subfamily members with gene specific primers were confirmed by quantitative RT-PCR. The values were normalized to *NbActin* and were calculated to the control. Values are means±SD (n = 3). Similar results were obtained from at least two experiments. One representative experiment is shown. Asterisks indicate significant differences relative to the control as determined by Student’s *t* test (*P<0.05, **P<0.01, ***P<0.001).
(TIF)

Figure S5 **Transcript levels of M24, S01 and M18 protease subfamily members in protease-silenced plants.** A. Transcript levels of M24 protease subfamily members were examined in *Ncn2132-*silenced plants. B. Transcript levels of M24 protease subfamily members were examined in *Ncn881*-silenced plants. C. Transcript levels of M24 protease subfamily members were examined in *Ncn9826-* silenced plants. D. Transcript level of S01 protease subfamily members was examined in *Ncn964*-silenced plants. E. Transcript level of M18 protease subfamily members was examined in *Ncn8326-*silenced plants. Values are means±SD (n = 3). The values were normalized to *NbActin* and were calculated to the control. Similar results were obtained from at least two experiments. One representative experiment is shown.
(TIF)

Table S1 **List of selected 153 proteases with its corresponding proteases from different organisms.** ^a^Classification abbreviations : (A) = Aspartic, (C) = Cysteine, (M) = Metallo-, (S) = Serine, (T) = Threonine proteases family based on MEROPS classification system (http://merops.sanger.ac.uk/). The corresponding proteases in the^ b^ *N. benthamiana* genome (http://solgenomics.net/, Niben.genome.v0.4.4), ^d^tomato genome (http://solgenomics.net/, ITAG2.40), ^e^potato genome (http://solgenomics.net/PGSC DM v3.4), ^f^ *Arabidopsis* genome (http://www.arabidopsis.org/, TAIR10) and ^g^other organims from the Genbank database (http://www.ncbi.nlm.nih.gov/genbank/). ^c^Numbers in () indicates the additional accession number which corresponds to the pepper EST ID.
(DOCX)

Table S2 **List of 153 protease-silenced plants with developmental phenotypes and responses to incompatible and compatible pathogen.** ^a^ Developmental phenotypes abbreviations : N.D. = No difference, ISG = Inhibition of shoot growth, ISG+ALS = Inhibition of shoot growth with abnormal leaf shape, L = Lethality, LCC = Leaf color changed, ALS = Abnormal leaf shape. ^b^Response to incompatible pathogens abbreviations :+ = Delayed HR, − = Enhanced HR, N.D. = No difference, L = Lethality. ^c^Response to compatible pathogens abbreviations :+ = Delayed symptom, N.D. = No difference, L = Lethality.
(DOCX)

Table S3 **Primer information for quantitative RT-PCR analysis.**
(DOCX)
