# Supplementary figures and images for: Multiple Classes of Immune-Related Proteases Associated with the Cell Death Response in Pepper Plants
Source: PLoS One. 2013 May 16;8(5):e63533. doi: 10.1371/journal.pone.0063533 (PMC3656034; doi:10.1371/journal.pone.0063533)

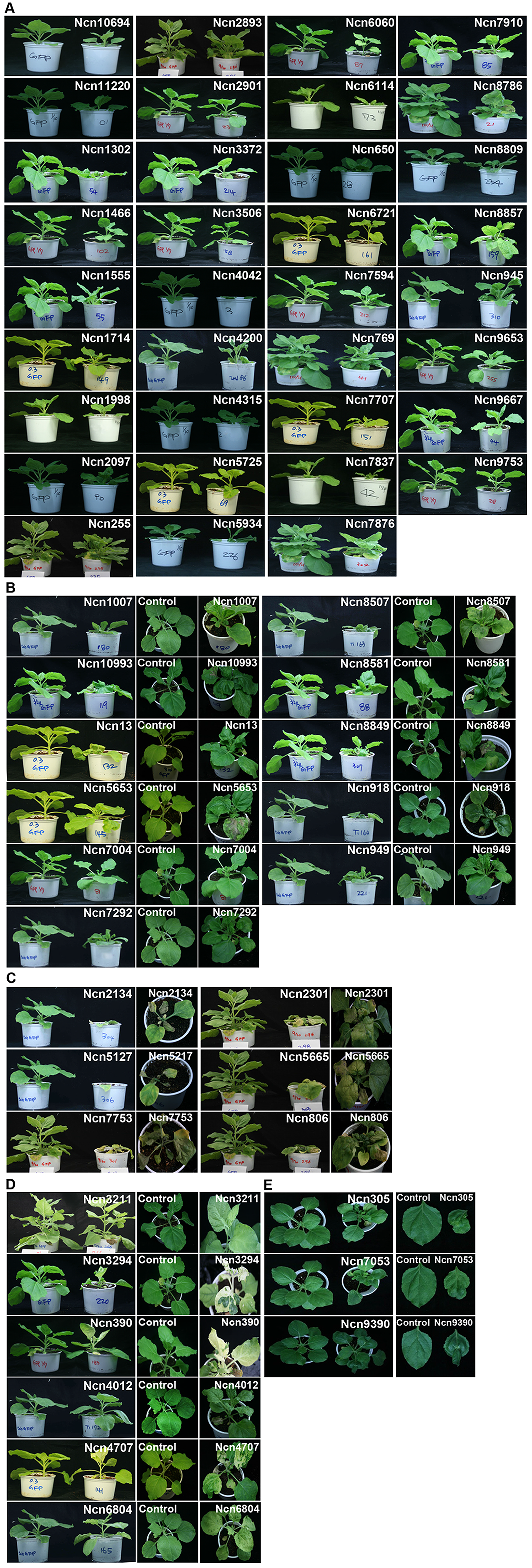

Supplement: Figure S1 — Altered phenotypes of protease-silenced plants. Protease-silenced plants showing altered phenotypes are categorized into 5 classes. The phenotypes are A. Inhibition of shoot growth. B. Inhibition of shoot growth with abnormal leaf shape. C. Lethality. D. Leaf color change. E. Crinkled leaves. These phenotype changes had been observed for 3 or 4 weeks and the picture are taken at 3 or 4 weeks after silencing. For every protease gene, 4 plants were silenced at each experiment. Similar results were obtained from at least three independent experiments. One representative experiment is shown. (TIF) [file pone.0063533.s001.tif]

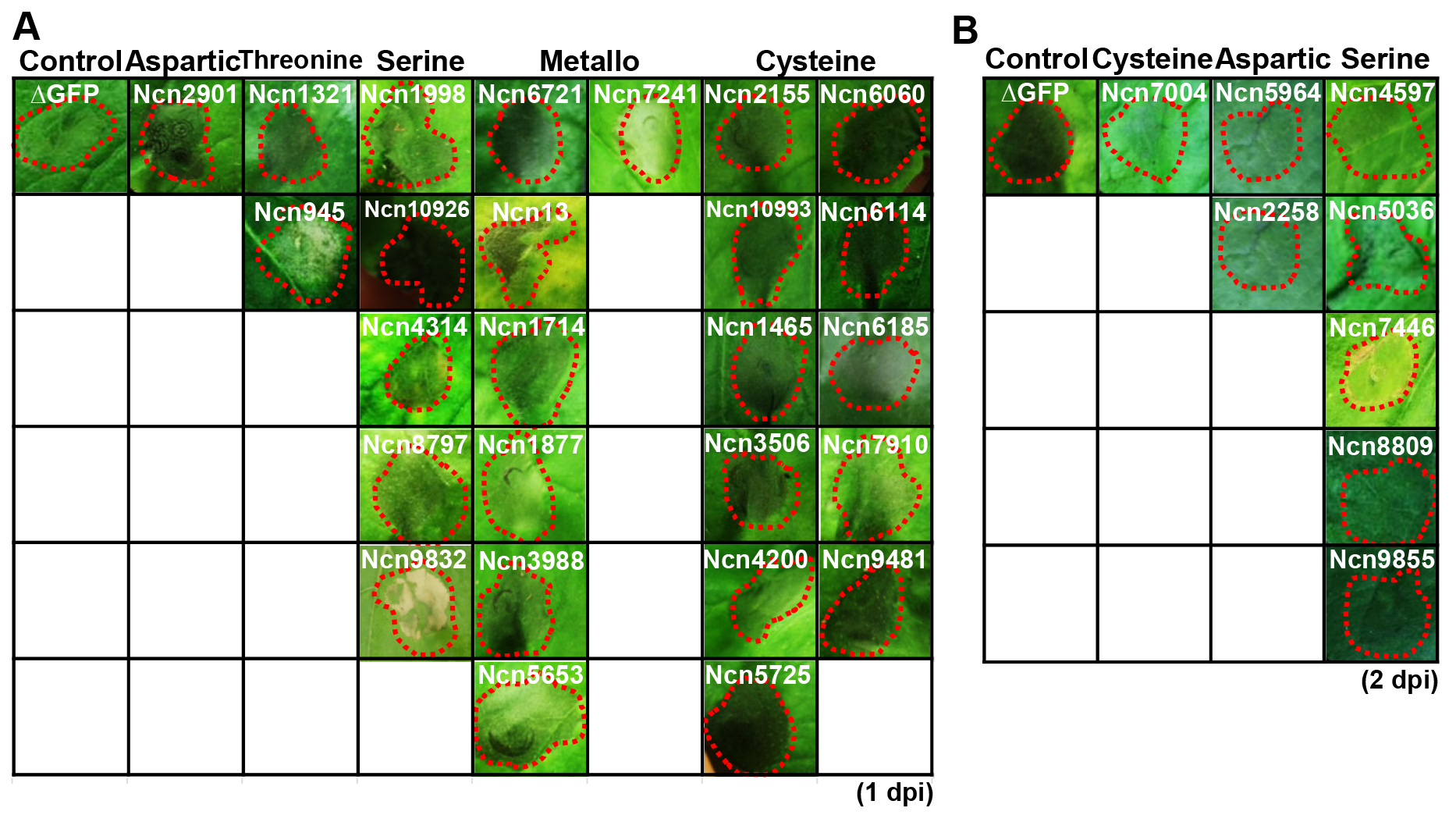

Supplement: Figure S2 — Enhanced and delayed HR responses in protease silenced-plant following incompatible pathogen infection. Protease-silenced plants were infiltrated with non-host bacterial pathogen P. syringae pv. tomato T1 (OD600 = 0.2). The HR cell death symptoms were taken at 1 dpi (A) and 2 dpi (B). The phenotypes indicate enhanced (A) or delayed HR (B) responses. For every protease gene, 6 sections per 1 leaf were infected with the pathogen and total 2 leaves were used for 1 plant. Total 4 plants were infiltrated at each experiment and done it at least three repeated tests. Red dotted line indicates the site of P. syringae pv. tomato T1 infection. One representative experiment is shown. (TIF) [file pone.0063533.s002.tif]

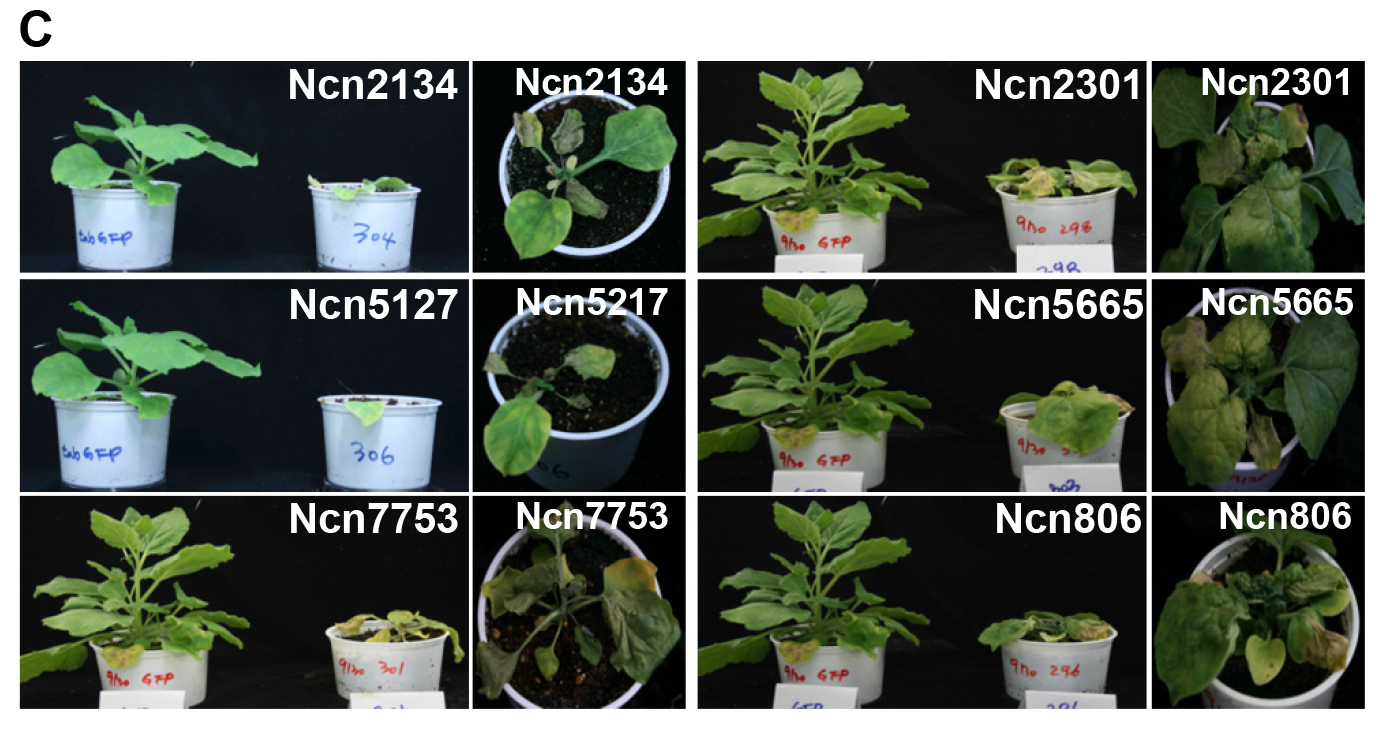

Supplement: Figure S3 — Delayed symptom development in protease-silenced plant following compatible pathogen infection. Protease-silenced plants were infiltrated with host bacterial pathogen P. syringae pv. tabaci (OD600 = 0.005). The disease symptoms induced by the pathogen were taken at 3 dpi. For every protease gene, 6 sections per 1 leaf were infected with the pathogen and total 2 leaves were used for 1 plant. Total 4 plants were infiltrated at each experiment and done it at least three repeated tests. Red dotted line indicates the site of P. syringae pv. tabaci infection. One representative experiment is shown. (TIF) [file pone.0063533.s003.tif]

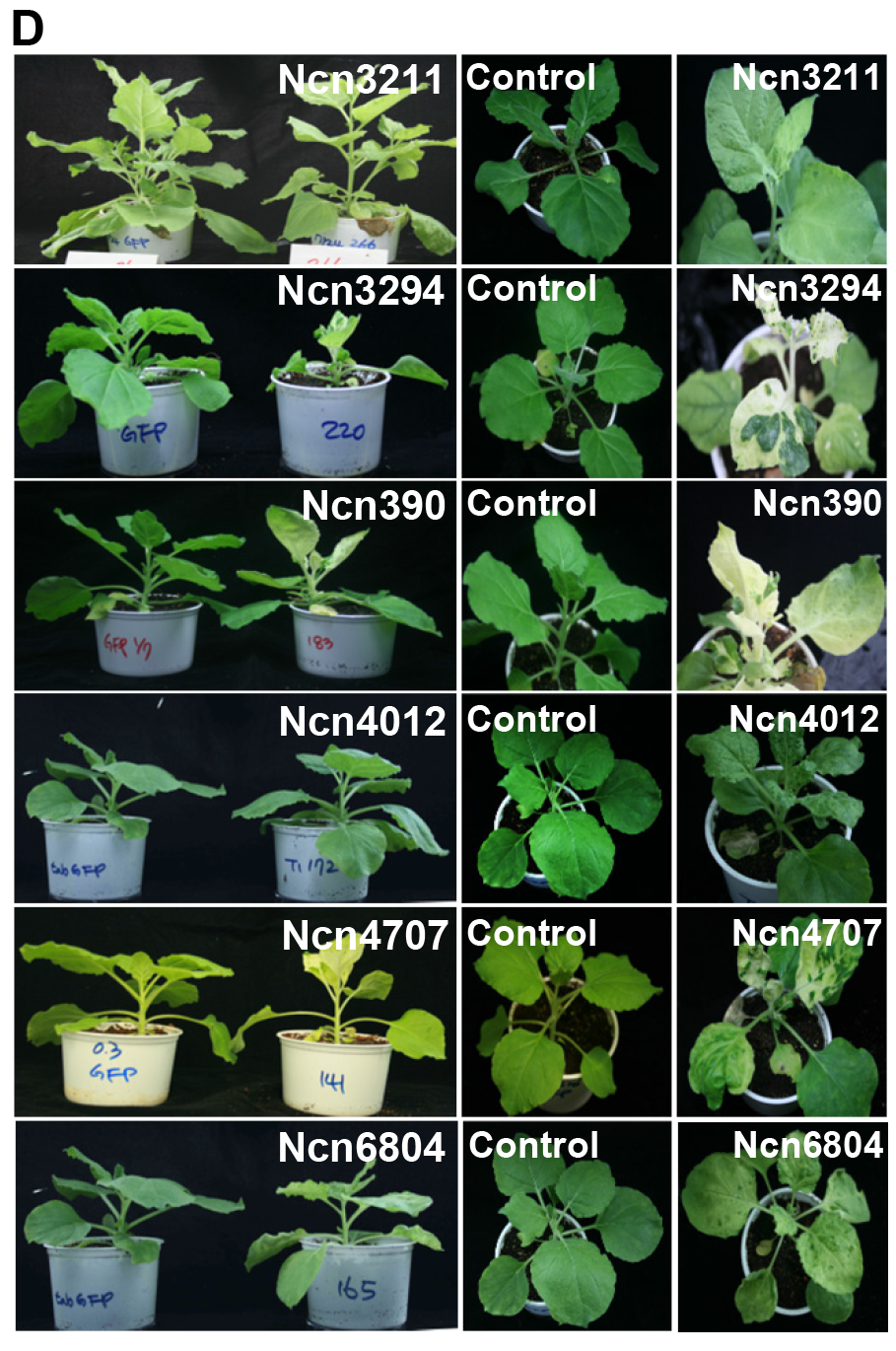

Supplement: Figure S4 — Confirmation of gene-specific silencing in M24, S01 and M18 proteases subfamily. Silencing confirmation of M24 (A), S01 (B) and M18 (C) protease subfamily members with gene specific primers were confirmed by quantitative RT-PCR. The values were normalized to NbActin and were calculated to the control. Values are means±SD (n = 3). Similar results were obtained from at least two experiments. One representative experiment is shown. Asterisks indicate significant differences relative to the control as determined by Student’s t test (*P<0.05, **P<0.01, ***P<0.001). (TIF) [file pone.0063533.s004.tif]

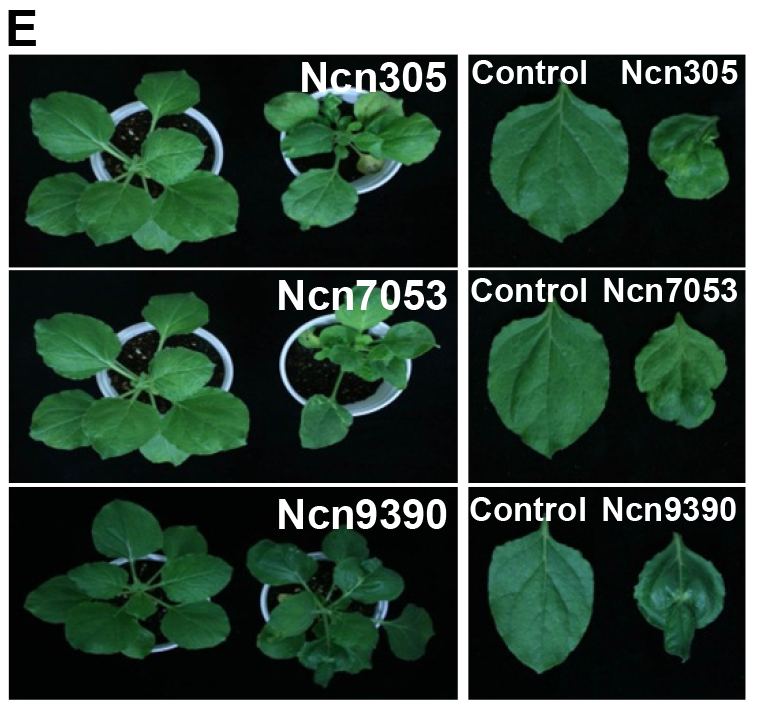

Supplement: Figure S5 — Transcript levels of M24, S01 and M18 protease subfamily members in protease-silenced plants. A. Transcript levels of M24 protease subfamily members were examined in Ncn2132-silenced plants. B. Transcript levels of M24 protease subfamily members were examined in Ncn881-silenced plants. C. Transcript levels of M24 protease subfamily members were examined in Ncn9826- silenced plants. D. Transcript level of S01 protease subfamily members was examined in Ncn964-silenced plants. E. Transcript level of M18 protease subfamily members was examined in Ncn8326-silenced plants. Values are means±SD (n = 3). The values were normalized to NbActin and were calculated to the control. Similar results were obtained from at least two experiments. One representative experiment is shown. (TIF) [file pone.0063533.s005.tif]
