# Supplementary material for: Multiple Classes of Immune-Related Proteases Associated with the Cell Death Response in Pepper Plants
Source: PLoS One. 2013 May 16;8(5):e63533. doi: 10.1371/journal.pone.0063533 (PMC3656034; doi:10.1371/journal.pone.0063533)
Supplement: Table S1 — List of selected 153 proteases with its corresponding proteases from different organisms. aClassification abbreviations : (A) = Aspartic, (C) = Cysteine, (M) = Metallo-, (S) = Serine, (T) = Threonine proteases family based on MEROPS classification system (http://merops.sanger.ac.uk/). The corresponding proteases in the b N. benthamiana genome (http://solgenomics.net/, Niben.genome.v0.4.4), dtomato genome (http://solgenomics.net/, ITAG2.40), epotato genome (http://solgenomics.net/PGSC DM v3.4), f Arabidopsis genome (http://www.arabidopsis.org/, TAIR10) and gother organims from the Genbank database (http://www.ncbi.nlm.nih.gov/genbank/). cNumbers in () indicates the additional accession number which corresponds to the pepper EST ID. (DOCX) [file pone.0063533.s006.docx]

## Supporting Information Tables

**Table S1. List of selected 153 proteases with its corresponding proteases from different organisms.**

|  | **Database annotation** | | | **Accession No. from** | | | | |
| --- | --- | --- | --- | --- | --- | --- | --- | --- |
| **Pepper EST ID** | **MEROPS ID (Classi-fication^a^)** | **Description** | **E-value** | ***N. benthamiana*^b^( )^c^** | ***S. lycopersicum*^d^** | ***S. phureja*^e^** | ***Arabidopsis*^f^** | **Genbank^g^** |
| Ncn1007 | MER003910 (M) | AtFtsH2 peptidase (*Capsicum annuum*) | 3.00E-99 | NbS00015215g0002.1 | Solyc07g055320.1.1 | PGSC0003DMG400017311 | AT2G30950 | AJ012165.1 |
| Ncn10366 | MER024778 (M) | indoleacetyl amino acid hydrolase (*Oryza sativa*) | 2.00E-59 | NbS00030956g0019.1 (2) | Solyc03g121270.2.1 | PGSC0003DMP400004648 |  | XP_003546640.1 |
| Ncn10583 | MER003372 (M) | At5g60160-like peptidase (*Arabidopsis thaliana*) | 6.00E-13 | NbS00031969g0005.1 | Solyc03g111180.1.1 | PGSC0003DMT400039280 | AT5G60160 | XM_002283439.1 |
| Ncn10694 | MER015561 (A) | At5g37540 (*Arabidopsis thaliana*) | 7.00E-58 | NbS00016717g0002.1 (3) | Solyc11g011440.1.1 | PGSC0003DMP400025733 | AT5G37540 | XP_002263620.2 |
| Ncn10706 | MER002771 (S) | P69 peptidase (*Solanum lycopersicum*) | 1.00E-130 | NbS00025163g0002.1 (1) | Solyc08g007610.1.1 | PGSC0003DMP400022145 |  | ABG37022.1 |
| Ncn10708 | MER000392 (S) | prolyl oligopeptidase (*Sus scrofa*) | 1.00E-11 | NbS00039059g0002.1 | Solyc04g082120.2.1 | PGSC0003DMP400017449 | AT1G76140 | ACG43067.1 |
| Ncn10742 | MER005899 (S) | P69 peptidase (*Solanum lycopersicum*) | 8.00E-89 | NbS00017912g0003.1 (4) | Solyc08g007700.1.1 | PGSC0003DMP400007010 | AT5G51750 | XP_003517445.1 |
| Ncn10829 | MER006019 (S) | At3g02110 (*Arabidopsis thaliana*) | 0 | NbS00034187g0001.1 | Solyc02g088820.2.1 | PGSC0003DMP400002640 | AT3G02110 | XP_002280058.1 |
| Ncn10926 | MER006067 (S) | At5g22860 (*Arabidopsis thaliana*) | 2.00E-49 | NbS00037462g0004.1 | Solyc11g066590.1.1 |  | AT5G22860 | XP_002271797.1 |
| Ncn10993 | MER055132 (C) | family C19 unassigned peptidases (*Oryza sativa*) | 1.00E-111 | NbS00016880g0009.1 (2) | Solyc05g055090.1.1 | PGSC0003DMP400005419 | AT3G11910 | ACJ04334.1 |
| Ncn1105 | MER005994 (S) | At5g24260 (*Arabidopsis thaliana*) | 5.00E-24 | NbS00002765g0024.1 |  | PGSC0003DMG400009126 | AT5G24260 | XP_002277892.2 |
| Ncn1106 | MER055153 (S) | At5g24260 (*Oryza sativa*) | 3.00E-58 | NbS00026455g0006.1 (1) | Solyc03g083330.2.1 | PGSC0003DMP400016071 | AT5G24260 | XP_003526712.1 |
| Ncn11088 | MER001368 (S) | LeSBT1 peptidase (*Arabidopsis thaliana*) | 1.00E-107 | NbS00009728g0003.1 (1) | Solyc03g044150.2.1 | PGSC0003DMP400037294 | AT2G05920 | ABQ58080.1 |
| Ncn11220 | MER014505 (A) | phytepsin (*Ipomoea batatas*) | 8.00E-19 | NbS00035939g0004.1 (1) | Solyc09g082760.2.1 | PGSC0003DMP400052543 | AT1G69100 | XP_003626142.1 |
| Ncn1175 | MER019084 (C) | RCR3 peptidase (*Solanum lycopersicum*) | 3.00E-97 | NbS00025385g0005.1 | Solyc02g077040.2.1 | PGSC0003DMP400018074 |  | NP_001233949.1 |
| Ncn13 | MER068105 (M) | family M41 unassigned peptidases (*Myxococcus xanthus*) | 5.00E-53 | NbS00048541g0015.1 (3) | Solyc01g109940.2.1 | PGSC0003DMP400053921 | AT1G45000 | XP_002283544.1 |
| Ncn1302 | MER014640 (C) | At2g24640 (*Arabidopsis thaliana*) | 2.00E-98 | NbS00027278g0001.1 (1) | Solyc08g069050.1.1 | PGSC0003DMC400045795 | AT2G24640 | XP_002515370.1 |
| Ncn1321 | MER020043 (T) | proteasome subunit alpha 3 (*Nicotiana tabacum*) | 1.00E-125 | NbS00028089g0021.1 (2) | Solyc10g077030.1.1 | PGSC0003DMP400012746 | AT2G27020 | XP_002283849.1 |
| Ncn1465 | MER004311 (C) | Ubp3 ubiquitin peptidase (plant-type) (*Arabidopsis thaliana*) | 2.00E-65 | NbS00005203g0016.1 (1) | Solyc01g105240.2.1 | PGSC0003DMP400022225 | AT4G39910 | XP_002270407.1 |
| Ncn1466 | MER034785 (C) | family C19 unassigned peptidases (*Oryza sativa*) | 1.00E-152 | NbS00003042g0016.1 | Solyc04g056290.2.1 | PGSC0003DMP400019660 | AT2G22310 | XP_003538616.1 |
| Ncn1555 | MER002266 (C) | subfamily C1A unassigned peptidases (*Pisum sativum*) | 4.00E-08 | NbC25499389g0001.1 | Solyc02g076980.2.1 | PGSC0003DMP400062943 | AT1G29080 | AAB41816.1 |
| Ncn1714 | MER005070 (M) | At1g24140 like peptidase (*Arabidopsis thaliana*) | 1.00E-88 | NbS00021642g0003.1 (1) | Solyc04g005040.1.1 | PGSC0003DMT400004843 | AT1G70170 | GU441835.1 |
| Ncn1750 | MER003443 (S) | Lon peptidase homologue (type 3) (*Spinacia oleracea*) | 7.00E-28 | NbS00042527g0008.1 | Solyc08g077440.2.1 | PGSC0003DMP400003299 | AT5G47040 | XP_002282657.1 |
| Ncn1877 | MER001949 (M) | leucyl aminopeptidase (plant-type) (*Solanum lycopersicum*) | 0 | NbS00027882g0011.1 | Solyc12g010040.1.1 | PGSC0003DMP400013800 | AT2G24200 | XP_003633175.1 |
| Ncn1929 | MER026245 (S) | family S28 non-peptidase homologues (*Oryza sativa*) | 1.00E-154 | NbS00017103g0005.1 (1) | Solyc03g033620.2.1 |  | AT4G36195 | XP_003612122.1 |
| Ncn1954 | MER015431 (S) | At1g32950 (*Arabidopsis thaliana*) | 7.00E-31 | NbS00009161g0010.1 | Solyc03g081260.2.1 | PGSC0003DMP400001181 | AT1G32950 | XP_002891020.1 |
| Ncn1998 | MER003532 (S) | prolyl aminopeptidase (*Arabidopsis thaliana*) | 1.00E-154 | NbS00039951g0005.1 | Solyc11g044310.1.1 |  | AT2G14260 | XM_002271253.2 |
| Ncn1999 | MER025953 (S) | prolyl aminopeptidase (*Prochlorococcus marinus*) | 7.00E-08 | NbS00039951g0010.1 | Solyc11g044310.1.1 |  | AT2G14260 | XP_002271289.2 |
| Ncn2083 | MER021438 (C) | family C2 unassigned peptidases (*Anopheles gambiae*) | 3.00E-16 | NbS00045815g0003.1 (2) | Solyc10g079890.1.1 | PGSC0003DMP400051974 | AT3G10300 | CAB63845.1 |
| Ncn2097 | MER031222 (C) | pseudotzain (*Helianthus annuus*) | 8.00E-19 | NbC26208175g0003.1 (2) | Solyc04g078540.2.1 | PGSC0003DMP400014071 | AT1G47128 | CAH59429.1 |
| Ncn2099 | MER005737 (C) | pseudotzain (*Solanum tuberosum*) | 0 | NbS00040506g0007.1 (1) | Solyc12g088670.1.1 | PGSC0003DMP400044879 | AT4G36880 | ADR32296.1 |
| Ncn2110 | MER015585 (A) | At1g77480 (*Arabidopsis thaliana*) | 8.00E-48 | NbS00031161g0003.1 (1) | Solyc06g009110.2.1 | PGSC0003DMG400014646 |  |  |
| Ncn2132 | MER027216 (M) | Xaa-Pro dipeptidase {eukaryote} (*Arabidopsis thaliana*) | 2.00E-16 | NbS00024034g0008.1 | Solyc07g026950.2.1 |  | AT4G29490 | XP_002514160.1 |
| Ncn2134 | MER001710 (T) | proteasome subunit beta 3 *(Nicotiana tabacum*) | 1.00E-106 | NbS00053635g0009.1 (3) | Solyc04g009410.2.1 | PGSC0003DMT400082475 | AT1G21720 | XM_002285835.2 |
| Ncn2155 | MER024771 (C) | At1g56700 ({Arabidopsis thaliana}) (*Oryza sativa*) | 1.00E-26 | NbS00016770g0011.1 (1) | Solyc01g100970.2.1 | PGSC0003DMP400005001 | AT1G56700 | XP_002280759.1 |
| Ncn2258 | MER001950 (A) | phytepsin (*Solanum lycopersicum*) | 1.00E-148 | NbS00031482g0006.1 (3) | Solyc01g101240.2.1 | PGSC0003DMP400031856 | AT1G62290 | ABB87123.1 |
| Ncn2301 | MER004206 (T) | proteasome subunit alpha 5 (*Glycine max*) | 1.00E-128 | NbS00045746g0008.1 (5) | Solyc02g070510.2.1 | PGSC0003DMT400054523 | AT1G53850 | XM_002265527.2 |
| Ncn2327 | MER015428 (S) | AtSBT1 peptidase (*Arabidopsis thaliana*) | 1.00E-31 | NbS00018081g0024.1 | Solyc01g091920.2.1 | PGSC0003DMP400000294 |  |  |
| Ncn2393 | MER002370 (C) | vignain *(Hemerocallis sp*.) | 4.00E-26 | NbS00019994g0012.1 (1) | Solyc07g053460.2.1 | PGSC0003DMP400016624 | AT3G48350 | XP_002525147.1 |
| Ncn2523 | MER012288 (M) | At5g42620 (*Arabidopsis thaliana*) | 4.00E-09 | NbS00031823g0010.1 | Solyc01g016990.1.1 |  | AT5G42620 | NP_568608.2 |
| Ncn255 | MER005714 (S) | At3g57680 (*Arabidopsis thaliana*) | 3.00E-57 | NbS00010528g0007.1 | Solyc02g071190.2.1 | PGSC0003DMP400056330 | AT3G57680 | NP_001148747.1 |
| Ncn259 | MER055218 (S) | subfamily S41A non-peptidase homologues (*Oryza sativa*) | 1.00E-170 | NbS00023562g0010.1 | Solyc03g059260.2.1 | PGSC0003DMG400033928 | AT4G17740 | XP_002277512.2 |
| Ncn2893 | MER006003 (S) | serine carboxypeptidase C (*Solanum lycopersicum*) | 1.00E-126 | NbS00001183g0003.1 | Solyc06g083030.2.1 | PGSC0003DMP400034903 | AT4G12910 | XP_002527263.1 |
| Ncn2901 | MER015556 (A) | At1g09750-type peptidase (*Arabidopsis thaliana*) | 2.00E-21 | NbS00004524g0008.1 (3) | Solyc12g087940.1.1 | PGSC0003DMG400002046 |  |  |
| Ncn2967 | MER011809 (M) | aminoacylase (*Arabidopsis thaliana*) | 2.00E-51 | NbS00000860g0010.1 | Solyc10g074580.1.1 | PGSC0003DMP400029396 |  |  |
| Ncn2971 | MER036035 (S) | acylaminoacyl-peptidase *(Arabidopsis thaliana*) | 0 | NbS00042064g0005.1 | Solyc03g005340.2.1 | PGSC0003DMP400023791 | AT4G14570 | NP_193193.2 |
| Ncn304 | MER043005 (S) | serine carboxypeptidase D (*Oryza sativa*) | 1.00E-119 | NbS00005982g0008.1 | Solyc01g108450.2.1 | PGSC0003DMG402025899 |  | XP_002285022.1 |
| Ncn305 | MER006305 (S) | family S10 unassigned peptidases (*Hordeum vulgare*) | 1.00E-44 | NbS00030940g0009.1 | Solyc01g108490.2.1 | PGSC0003DMP400044934 |  |  |
| Ncn3211 | MER001381 (S) | peptidase Clp (type 6) (*Solanum lycopersicum*) | 1.00E-120 | NbS00015416g0017.1 (1) | Solyc10g051310.1.1 | PGSC0003DMP400056311 | AT1G11750 | XP_003537887.1 |
| Ncn3294 | MER006063 (S) | ClpP4 peptidase (plant) (*Arabidopsis thaliana*) | 1.00E-93 | NbS00017607g0008.1 (1) | Solyc08g075750.2.1 | PGSC0003DMG400030851 | AT5G45390 | XP_003534318.1 |
| Ncn3372 | MER013917 (S) | At5g36180 (*Arabidopsis thaliana*) | 4.00E-23 | NbS00058916g0004.1 | Solyc05g050780.2.1 | PGSC0003DMP400012453 | AT5G36180 | XP_002269439.1 |
| Ncn3373 | MER015618 (S) | At1g73290 (*Arabidopsis thaliana*) | 6.00E-42 | NbS00024397g0006.1 | Solyc05g050770.2.1 | PGSC0003DMP400012456 | AT1G73300 | XP_002510077.1 |
| Ncn3506 | MER013524 (C) | ubiquitin-specific peptidase 7 (*Arabidopsis thaliana*) | 2.00E-85 | NbS00012989g0120.1 (3) | Solyc05g055090.2.1 | PGSC0003DMG402003010 | AT5G06600 | FJ264198.1 |
| Ncn3606 | MER067801 (S) | SCO7095-type peptidase (*Mesorhizobium sp. BNC1*) | 4.00E-13 | NbS00008723g0009.1 (1) | Solyc03g083010.1.1 | PGSC0003DMG401018530 | AT5G53050 | XP_002865953.1 |
| Ncn390 | MER001519 (S) | peptidase Clp (type 1) (*Solanum lycopersicum*) | 1.00E-143 | NbS00010569g0102.1 (2) | Solyc01g100520.2.1 | PGSC0003DMP400004968 | AT1G02560 | ABA41488.1 |
| Ncn3963 | MER015620 (S) | At1g06870 (*Arabidopsis thaliana*) | 2.00E-08 | NbS00001712g0020.1 | Solyc04g079550.2.1 | PGSC0003DMP400020703 | AT1G06870 |  |
| Ncn3988 | MER036323 (M) | family M41 unassigned peptidases (*Solanum lycopersicum*) | 1.00E-174 | NbS00033574g0005.1 (1) | Solyc08g063050.2.1 | PGSC0003DMP400014432 | AT2G26140 | XP_002279005.2 |
| Ncn3992 | MER003940 (S) | plant mitochondrial rhomboid (*Arabidopsis thaliana*) | 8.00E-35 | NbS00062086g0005.1 | Solyc08g068270.2.1 | PGSC0003DMP400025580 | AT5G25752 | XP_002281548.1 |
| Ncn3994 | MER005991 (S) | oligopeptidase B (*Arabidopsis thaliana*) | 0 | NbS00008029g0007.1 | Solyc02g050260.1.1 | PGSC0003DMT400026141 | AT1G50380 | XM_003633018.1 |
| Ncn4012 | MER022428 (M) | leucyl aminopeptidase {animal} (*Anopheles gambiae*) | 2.00E-22 | NbS00024531g0008.1 |  |  |  | XP_003220494.1 |
| Ncn4042 | MER013896 (S) | At1g20160 (*Arabidopsis thaliana*) | 3.00E-36 | NbS00032404g0010.1 | Solyc01g006660.1.1 | PGSC0003DMG400021348 | AT1G20160 | XP_003523395.1 |
| Ncn4099 | MER006046 (S) | At5g23140-type peptidase (*Arabidopsis thaliana*) | 9.00E-95 | NbS00023281g0008.1 (2) | Solyc04g009310.2.1 | PGSC0003DMG402010580 | AT5G23140 | XP_002277069.1 |
| Ncn4141 | MER015694 (M) | At5g42320 (*Arabidopsis thaliana*) | 7.00E-78 | NbS00002313g0025.1 (1) | Solyc04g082340.2.1 | PGSC0003DMP400017620 |  | XP_002270277.2 |
| Ncn4199 | MER013567 (C) | At2g44140 (*Arabidopsis thaliana*) | 1.00E-58 | NbS00052496g0024.1 | Solyc01g006230.2.1 | PGSC0003DMG400032144 | AT2G44140 | NP_973687.1 |
| Ncn4200 | MER013565 (C) | At3g59950 (*Arabidopsis thaliana*) | 6.00E-97 | NbS00044895g0007.1 | Solyc01g006230.1.1 | PGSC0003DMT400081878 | AT3G59950 | XP_003635099.1 |
| Ncn4314 | MER004684 (S) | At2g23010 (*Arabidopsis thaliana*) | 2.00E-13 | NbS00036239g0006.1 | Solyc12g088280.1.1 | PGSC0003DMG400011542 | AT2G23010 |  |
| Ncn4315 | MER011747 (S) | At2g22920 (*Arabidopsis thaliana*) | 1.00E-131 | NbS00059561g0001.1 | Solyc04g077650.2.1 |  | AT2G22920 |  |
| Ncn4597 | MER022929 (S) | serine carboxypeptidase III (plant) (*Theobroma cacao*) | 1.00E-153 | NbS00017915g0109.1 | Solyc11g008280.1.1 | PGSC0003DMP400054112 | AT3G10410 | XP_002529593.1 |
| Ncn4707 | MER002008 (M) | chloroplast {stromal} processing peptidase (*Pisum sativum*) | 3.00E-97 | NbS00026030g0004.1 | Solyc03g118430.2.1 | PGSC0003DMP400025109 | AT5G42390 | XP_003610819.1 |
| Ncn4906 | MER005440 (C) | At4g24560 (*Arabidopsis thaliana*) | 1.00E-100 | NbS00005704g0004.1 (1) | Solyc04g076210.2.1 |  | AT4G24560 | BAD09009.1 |
| Ncn5036 | MER014317 (S) | At5g40200 (*Arabidopsis thaliana*) | 3.00E-58 | NbS00055986g0001.1 (3) | Solyc12g026400.1.1 | PGSC0003DMP400033658 | AT5G40200 | XP_002275131.1 |
| Ncn5127 | MER015628 (T) | proteasome subunit alpha 2 (*Arabidopsis thaliana*) | 1.00E-121 | NbS00002171g0114.1 (3) | Solyc10g008010.2.1 | PGSC0003DMP400018645 | AT1G16470 | XP_003516906.1 |
| Ncn5180 | MER005431 (C) | At4g17890 (*Arabidopsis thaliana*) | 4.00E-67 | NbS00020038g0002.1 (2) | Solyc08g076920.2.1 | PGSC0003DMG400017515 | AT4G17890 | XP_002278066.2 |
| Ncn5328 | MER000431 (S) | prolyl aminopeptidase (*Neisseria gonorrhoeae*) | 4.00E-35 | NbS00004854g0005.1 (1) | Solyc08g022070.2.1 | PGSC0003DMG400028869 | AT1G20380 | AEX58649.1 |
| Ncn5653 | MER042994 (M) | family M8 unassigned peptidases (*Oryza sativa*) | 3.00E-82 | NbS00012786g0007.1 | Solyc04g079900.2.1 | PGSC0003DMG400016879 | AT5G42620 | XP_002510341.1 |
| Ncn5665 | MER055029 (T) | proteasome subunit alpha 4 (*Solanum tuberosum*) | 1.00E-109 | NbS00016087g0008.1 (6) | Solyc02g081700.1.1 | PGSC0003DMT400049388 | AT3G22110 | AJ291733.1 |
| Ncn5725 | MER045657 (C) | cathepsin B, plant form (*Solanum tuberosum*) | 1.00E-180 | NbS00018589g0007.1 (3) | Solyc02g069100.2.1 | PGSC0003DMP400018307 | AT1G02305 | AY450641.1 |
| Ncn5885 | MER006049 (S) | At3g14240 (*Arabidopsis thaliana*) | 3.00E-69 | NbS00021903g0002.1 | Solyc03g123490.1.1 | PGSC0003DMP400016092 | AT3G14240 | XP_003632775.1 |
| Ncn5934 | MER005579 (S) | serine carboxypeptidase III (plant) (*Matricaria chamomilla*) | 1.00E-104 | NbS00019389g0006.1 | Solyc11g066250.1.1 | PGSC0003DMP400000884 | AT5G22980 | XP_002523707.1 |
| Ncn5964 | MER014522 (A) | At3g20015-type peptidase *(Arabidopsis thaliana*) | 2.00E-55 | NbS00059099g0001.1 | Solyc00g005000.2.1 | PGSC0003DMP400003920 | AT3G20015 | XP_002267930.1 |
| Ncn6037 | MER015632 (S) | AtRBL10 protein (*Arabidopsis thaliana*) | 2.00E-32 | NbS00015718g0023.1 (1) | Solyc01g104250.2.1 | PGSC0003DMP400041111 | AT1G25290 |  |
| Ncn6060 | MER027211 (C) | legumain {plant alpha form} (*Nicotiana tabacum*) | 0 | NbS00038670g0006.1 (6) | Solyc12g095910.1.1 | PGSC0003DMT400005630 |  | AJ841791.1 |
| Ncn6073 | MER010959 (S) | At1g33540 (*Arabidopsis thaliana*) | 4.00E-55 | NbS00017399g0020.1 | Solyc12g019560.1.1 | PGSC0003DMP400050768 | AT1G33540 |  |
| Ncn6114 | MER015602 (C) | glycosylphosphatidylinositol:protein transamidase (*Arabidopsis thaliana*) | 1.00E-59 | NbS00011726g0015.1 | Solyc10g007850.2.1 | PGSC0003DMP400036789 | AT1G08750 | NP_563825.1 |
| Ncn6185 | MER063900 (C) | metacaspase-9 ({Arabidopsis}-type) (*Medicago truncatula*) | 5.00E-64 | NbS00037357g0009.1 (1) | Solyc10g081300.1.1 | PGSC0003DMP400049028 |  |  |
| Ncn6260 | MER014085 (S) | At3g14067 (*Arabidopsis thaliana*) | 1.00E-152 | NbS00005824g0001.1 (1) | Solyc06g062950.1.1 | PGSC0003DMP400008705 | AT3G14067 | XP_003627323.1 |
| Ncn642 | MER036400 (S) | subfamily S26A unassigned peptidases (*Sorghum bicolor*) | 3.00E-41 | NbS00004167g0006.1 (1) | Solyc02g068390.2.1 | PGSC0003DMP400012556 | AT1G53530 | XP_002283744.1 |
| Ncn650 | MER004654 (S) | family S10 non-peptidase homologues (*Arabidopsis thaliana*) | 6.00E-47 | NbS00061295g0004.1 | Solyc04g077630.2.1 | PGSC0003DMP400022862 |  |  |
| Ncn651 | MER004653 (S) | At2g22970 (*Arabidopsis thaliana*) | 1.00E-127 | NbS00010416g0001.1 | Solyc04g076120.2.1 | PGSC0003DMP400022863 | AT2G22970 | XP_003593861.1 |
| Ncn652 | MER015653 (S) | At3g12203 (*Arabidopsis thaliana*) | 1.00E-139 | NbS00002902g0006.1 | Solyc04g077640.2.1 | PGSC0003DMP400018778 | AT3G12203 | XP_002283413.1 |
| Ncn665 | MER005524 (S) | SlSBT3 g.p. (*Solanum lycopersicum*) | 0 | NbS00028101g0006.1 (2) | Solyc01g087820.2.1 | PGSC0003DMT400017631 |  | DQ066722.1 |
| Ncn6706 | MER063993 (S) | family S24 unassigned peptidases (*Desulfohalobium retbaense*) | 8.00E-08 | NbS00036601g0004.1 (1) | Solyc03g117950.2.1 | PGSC0003DMP400025511 | AT5G51070 | XP_002283802.2 |
| Ncn6721 | MER015280 (M) | methionyl aminopeptidase 2 (*Arabidopsis thaliana*) | 1.00E-115 | NbS00011972g0104.1 (1) | Solyc01g006800.2.1 | PGSC0003DMG400021374 | AT3G59990 | XP_002270461.1 |
| Ncn6804 | MER015215 (M) | At3g27110 ({Arabidopsis thaliana}) (*Arabidopsis thaliana*) | 8.00E-11 | NbS00019551g0012.1 | Solyc11g068820.1.1 | PGSC0003DMG400013378 | AT3G27110 | XP_002509518.1 |
| Ncn6818 | MER014328 (S) | Mername-AA210 peptidase (*Arabidopsis thaliana*) | 1.00E-111 | NbS00026992g0027.1 | Solyc02g071560.2.1 | PGSC0003DMP400033261 |  | XP_002269555.2 |
| Ncn6835 | MER015123 (M) | chloroplast {stromal} processing peptidase (*Arabidopsis thaliana*) | 1.00E-125 | NbS00000278g0004.1 | Solyc03g118430.2.1 | PGSC0003DMG400010625 | AT5G56730 | AAA81472.1 |
| Ncn7004 | MER001021 (C) | At5g38200 (*Arabidopsis thaliana*) | 1.00E-74 | NbS00007507g0016.1 (3) | Solyc02g086300.1.1 | PGSC0003DMC400045795 | AT1G66860 |  |
| Ncn7053 | MER014076 (T) | proteasome subunit alpha 5 (*Arabidopsis thaliana*) | 8.00E-06 | NbS00000017g0011.1 |  | PGSC0003DMP400048702 |  |  |
| Ncn707 | MER000416 (S) | serine carboxypeptidase D (*Triticum aestivum*) | 1.00E-174 | NbS00000440g0112.1 | Solyc04g015340.2.1 | PGSC0003DMP400019834 | AT4G30810 | XP_002273324.1 |
| Ncn708 | MER029447 (S) | serine carboxypeptidase D (*Oryza sativa*) | 4.00E-26 | NbS00035448g0004.1 | Solyc07g005960.2.1 |  |  |  |
| Ncn7241 | MER015170 (M) | At1g67690-like peptidase (*Arabidopsis thaliana*) | 8.00E-65 | NbS00005259g0004.1 | Solyc05g014310.2.1 | PGSC0003DMP400030492 | AT1G67700 | XP_002515011.1 |
| Ncn7277 | MER005999 (S) | At3g45010 (*Arabidopsis thaliana*) | 2.00E-77 | NbS00018169g0009.1 (1) | Solyc05g041540.2.1 | PGSC0003DMP400000883 | AT3G45010 | CAC86383.1 |
| Ncn7288 | MER016204 (T) | proteasome subunit alpha 6 (*Nicotiana tabacum*) | 3.00E-09 | NbS00043198g0009.1 (2) | Solyc12g009140.1.1 | PGSC0003DMP400005232 | AT2G05840 | XP_002516258.1 |
| Ncn7292 | MER056043 (C) | At5g22030 (*Arabidopsis thaliana*) | 1.00E-103 | NbS00037871g0005.1 | Solyc06g073930.2.1 |  |  |  |
| Ncn7295 | MER023131 (M) | At1g48790-type peptidase (*Arabidopsis thaliana*) | 1.00E-103 | NbS00015181g0003.1 | Solyc01g060280.2.1 | PGSC0003DMP400009940 | AT1G48790 | XP_003620442.1 |
| Ncn7368 | MER054317 (S) | AIR3 peptidase (*Glycine max*) | 1.00E-141 | NbS00033716g0002.1 (1) | Solyc07g056170.2.1 |  |  | XP_002266728.1 |
| Ncn7420 | MER047630 (A) | subfamily A1B unassigned peptidases (*Medicago truncatula*) | 2.00E-62 | NbS00016801g0003.1 (1) | Solyc08g078670.2.1 | PGSC0003DMP400008524 | AT4G16563 | ACL81165.1 |
| Ncn7446 | MER030333 (S) | At2g19170/At4g30020/At2g19170 (*Lotus corniculatus*) | 1.00E-172 | NbS00045701g0004.1 (1) | Solyc07g008900.2.1 | PGSC0003DMG400028737 | AT4G30020 | XP_002269786.1 |
| Ncn75 | MER047519 (C) | metacaspase-1 ({Arabidopsis}-type) (*Medicago truncatula*) | 4.00E-08 | NbS00025677g0026.1 | Solyc01g105320.2.1 | PGSC0003DMG400012553 | AT1G02170 | XP_003547635.1 |
| Ncn7594 | MER026244 (S) | PIM1 peptidase (*Oryza sativa*) | 1.00E-108 | NbS00026887g0005.1 (1) | Solyc10g024320.1.1 |  | AT5G26860 | XP_002277956.1 |
| Ncn769 | MER013569 (A) | impas 1 peptidase (*Arabidopsis thaliana*) | 1.00E-152 | NbS00024101g0016.1 | Solyc01g008820.2.1 | PGSC0003DMP400039389 | AT2G03120 | XP_002875188.1 |
| Ncn7704 | MER000762 (S) | LeSBT1 peptidase (*Solanum lycopersicum*) | 2.00E-11 | NbS00054116g0012.1 (3) | Solyc04g078110.1.1 | PGSC0003DMP400026165 | AT5G67360 | XP_002283279.2 |
| Ncn7707 | MER011120 (M) | AtFtsH7 peptidase (*Arabidopsis thaliana*) | 1.00E-105 | NbS00037800g0004.1 | Solyc06g005950.2.1 | PGSC0003DMP400007417 | AT3G47060 | XP_003518301.1 |
| Ncn7737 | MER000764 (S) | P69 peptidase (*Solanum lycopersicum*) | 1.00E-149 | NbS00060293g0008.1 (4) | Solyc08g079840.1.1 | PGSC0003DMP400006965 |  | CAA07250.1 |
| Ncn7753 | MER006148 (T) | proteasome subunit beta 1 (*Petunia hybrida*) | 1.00E-118 | NbS00017089g0013.1 (3) | Solyc02g084920.2.1 | PGSC0003DMC400006278 | AT3G60820 | AJ291742.1 |
| Ncn7837 | MER015557 (A) | At2g39710-type peptidase (*Arabidopsis thaliana*) | 5.00E-97 | NbS00032080g0002.1 | Solyc02g065050.1.1 | PGSC0003DMP400044172 | AT2G39710 | XP_002283126.1 |
| Ncn7876 | MER054577 (M) | family M79 unassigned peptidases | 1.00E-159 | NbS00037461g0006.1 | Solyc05g007500.2.1 | PGSC0003DMP400054488 | AT1G14270 | XP_003530698.1 |
| Ncn7910 | MER005401 (C) | bacteriocin-processing peptidase (*Enterococcus faecium*) | 5.00E-06 | NbS00004419g0001.1 (2) | Solyc02g068180.2.1 |  |  | XP_002512910.1 |
| Ncn806 | MER004325 (T) | proteasome catalytic subunit 2 (*Arabidopsis thaliana*) | 1.00E-131 | NbS00022575g0009.1 (3) | Solyc05g013820.2.1 | PGSC0003DMG400016969 | AT5G40580 | AJ291736.1 |
| Ncn8210 | MER005676 (S) | OsBISCPL1-type putative carboxypeptidase (*Arabidopsis thaliana*) | 3.00E-90 | NbS00028196g0002.1 | Solyc11g066520.1.1 | PGSC0003DMG400000422 | AT2G27920 | XP_002273519.1 |
| Ncn8285 | MER063464 (S) | phytaspase (*Medicago truncatula*) | 2.00E-34 | NbS00004957g0121.1 | Solyc04g078740.2.1 | PGSC0003DMT400020546 |  | ACT34764.1 |
| Ncn8326 | MER015303 (M) | At5g04710 (*Arabidopsis thaliana*) | 1.00E-154 | NbS00026037g0013.1 (1) | Solyc11g007090.1.1 | PGSC0003DMP400026650 | AT5G04710 | XP_003591777.1 |
| Ncn8350 | MER044899 (S) | At1g74280 (*Arabidopsis thaliana*) | 1.00E-111 | NbS00059475g0001.1 (3) | Solyc09g009520.2.1 | PGSC0003DMP400004760 | AT1G74280 |  |
| Ncn8392 | MER029625 (S) | subfamily S8A unassigned peptidases (*Nicotiana tabacum*) | 0 | NbS00026520g0006.1 | Solyc02g068280.2.1 | PGSC0003DMP400016639 |  | XP_002263237.2 |
| Ncn8507 | MER049897 (M) | FtsH peptidase ({Thermotoga}-type) (*Moorella thermoacetica*) | 3.00E-44 | NbS00009697g0013.1 (3) | Solyc01g068330.1.1 | PGSC0003DMT400045722 |  |  |
| Ncn8581 | MER005448 (C) | AtUBP5 peptidase-like peptidase (*Arabidopsis thaliana*) | 4.00E-41 | NbS00017309g0123.1 (1) | Solyc09g019970.1.1 | PGSC0003DMT400031871 | AT2G40930 | XP_002881749.1 |
| Ncn8762 | MER029064 (C) | otubain-1 (*Arabidopsis thaliana*) | 1.00E-107 | NbS00028970g0025.1 | Solyc04g045480.2.1 | PGSC0003DMP400011835 | AT1G28120 | ABU93349.1 |
| Ncn8786 | MER015560 (A) | At5g22850-type peptidase (*Arabidopsis thaliana*) | 2.00E-87 | NbS00053010g0005.1 (1) | Solyc11g066620.1.1 | PGSC0003DMG401000428 | AT5G22850 | XP_002272121.1 |
| Ncn8797 | MER016676 (S) | serine carboxypeptidase C (Oryza sativa) | 5.00E-40 | NbS00028915g0023.1 | Solyc04g077670.2.1 | PGSC0003DMP400018772 |  |  |
| Ncn8809 | MER017373 (S) | At4g24760 (*Arabidopsis thaliana*) | 1.00E-100 | NbS00032670g0019.1 (6) | Solyc03g111770.2.1 | PGSC0003DMP400046159 | AT4G24760 | XP_002269274.1 |
| Ncn881 | MER015285 (M) | proliferation-association protein 1 (*Oryza sativa*) | 5.00E-39 | NbS00006055g0014.1 (3) | Solyc06g082090.2.1 | PGSC0003DMP400052850 | AT3G51800 | XP_003516956.1 |
| Ncn8815 | MER026248 (S) | RBL2 peptidase (*Oryza sativa*) | 2.00E-32 | NbS00033479g0016.1 (1) | Solyc08g080500.2.1 | PGSC0003DMP400005480 |  | ABG66197.1 |
| Ncn8849 | MER005388 (T) | proteasome subunit alpha 7 (*Cicer arietinum*) | 1.00E-21 | NbS00021500g0008.1 (3) | Solyc01g111450.1.1 | PGSC0003DMT400015849 | AT3G51260 | DQ226996.1 |
| Ncn8857 | MER015193 (M) | AtFtsH6 peptidase (*Arabidopsis thaliana*) | 1.00E-107 | NbC25032573g0001.1 | Solyc02g081550.2.1 | PGSC0003DMG400017730 | AT5G15250 | XP_003552529.1 |
| Ncn8884 | MER027250 (A) | At1g63690 (*Arabidopsis thaliana*) | 1.00E-116 | NbS00003467g0023.1 (4) | Solyc08g081180.2.1 | PGSC0003DMP400021838 | AT1G63690 | XP_003521881.1 |
| Ncn8903 | MER014056 (M) | PreP peptidase (Arabidopsis thaliana) | 0 | NbS00011015g0025.1 | Solyc01g108600.2.1 | PGSC0003DMP400044756 | AT3G19170 | XP_003636021.1 |
| Ncn9014 | MER015682 (S) | tyrosyl aminopeptidase ({Raphanus}-type) (*Arabidopsis thaliana*) | 0 | NbS00029012g0013.1 (1) | Solyc04g049630.2.1 | PGSC0003DMG400008244 |  | BAD35316.1 |
| Ncn918 | MER047647 (M) | PSMD14 peptidase (*Medicago truncatula*) | 1.00E-158 | NbS00040618g0005.1 (1) | Solyc04g079200.2.1 | PGSC0003DMP400014118 | AT5G23540 | XP_002284566.1 |
| Ncn9260 | MER034831 (S) | family S9 unassigned peptidases (*Oryza sativa*) | 1.00E-38 | NbS00024728g0003.1 (4) | Solyc08g076350.2.1 | PGSC0003DMG400006920 | AT1G32190 | XP_002518992.1 |
| Ncn9390 | MER015589 (A) | At1g49050-type peptidase (*Arabidopsis thaliana*) | 3.00E-53 | NbS00031201g0007.1 | Solyc06g069190.2.1 | PGSC0003DMG400004069 |  |  |
| Ncn945 | MER004348 (T) | proteasome subunit beta 4 (*Arabidopsis thaliana*) | 1.00E-116 | NbS00024067g0008.1 (4) | Solyc11g069150.1.1 | PGSC0003DMP400014198 | AT1G56450 | XP_002284989.1 |
| Ncn9481 | MER054716 (C) | At5g50260-type peptidase (*Nicotiana tabacum*) | 1.00E-174 | NbS00032670g0016.1 (3) | Solyc03g111730.2.1 | PGSC0003DMP400026598 | AT5G50260 | XP_002278323.1 |
| Ncn949 | MER003269 (S) | thylakoidal processing peptidase (*Arabidopsis thaliana*) | 2.00E-57 | NbS00012651g0209.1 | Solyc12g007120.1.1 | PGSC0003DMP400020703 |  |  |
| Ncn964 | MER050307 (S) | subfamily S1B unassigned peptidases (*Oryza sativa*) | 4.00E-14 | NbS00023513g0009.1 | Solyc02g082720.2.1 | PGSC0003DMP400025772 | AT5G27660 |  |
| Ncn9653 | MER000423 (S) | serine carboxypeptidase III (plant) (*Oryza sativa*) | 1.00E-143 | NbS00015028g0005.1 | Solyc06g074850.2.1 | PGSC0003DMP400012644 | AT1G15000 | NP_001045667.1 |
| Ncn9667 | MER019709 (A) | At1g05820 (*Arabidopsis thaliana*) | 5.00E-50 | NbS00032645g0008.1 (2) | Solyc09g098200.2.1 | PGSC0003DMP400021097 | AT1G05820 | XP_003535798.1 |
| Ncn9753 | MER005352 (A) | CND41 peptidase (*Nicotiana tabacum*) | 7.00E-32 | NbS00024574g0006.1 (1) | Solyc08g067100.2.1 | PGSC0003DMG400023144 | AT5G10760 |  |
| Ncn9826 | MER016542 (M) | At3g05350-type peptidase (*Arabidopsis thaliana*) | 5.00E-61 | NbS00009510g0004.1 (1) | Solyc01g099110.2.1 | PGSC0003DMP400042852 | AT3G05350 | XP_003554659.1 |
| Ncn9832 | MER005756 (S) | At4g10550 (*Arabidopsis thaliana*) | 2.00E-31 | NbS00003910g0012.1 | Solyc03g081250.1.1 |  | AT4G10550 | XP_002278574.2 |
| Ncn9846 | MER014000 (A) | phytepsin (*Nepenthes alata*) | 5.00E-89 | NbS00009953g0012.1 | Solyc02g032940.2.1 | PGSC0003DMP400038145 | AT4G04460 | XP_002279049.1 |
| Ncn9855 | MER015433 (S) | At1g32970 (*Arabidopsis thaliana*) | 9.00E-24 | NbS00032721g0002.1 |  | PGSC0003DMP400043762 | AT1G32970 |  |
| Ncn9856 | MER014089 (S) | At5g11940 (*Arabidopsis thaliana*) | 7.00E-27 | NbS00061770g0001.1 | Solyc05g013590.1.1 | PGSC0003DMP400032409 | AT5G11940 | XP_003629621.1 |
| Ncn9857 | MER015430 (S) | At1g32940 (*Arabidopsis thaliana*) | 3.00E-90 | NbS00011047g0007.1 |  | PGSC0003DMP400033695 | AT1G32940 |  |

^a^ Classification abbreviations : (A) = Aspartic, (C) = Cysteine, (M) = Metallo-, (S) = Serine, (T) = Threonine proteases family based on MEROPS classification system (http://merops.sanger.ac.uk/).

The corresponding proteases in the ^b^*N. benthamiana* genome (http://solgenomics.net/, Niben.genome.v0.4.4), ^d^tomato genome (http://solgenomics.net/, ITAG2.40), ^e^potato genome (http://solgenomics.net/PGSC DM v3.4), ^f^*Arabidopsis* genome (http://www.arabidopsis.org/, TAIR10) and ^g^other organims from the Genbank database (http://www.ncbi.nlm.nih.gov/genbank/).

^c^ Numbers in ( ) indicates the additional accession number which corresponds to the pepper EST ID.
