# Supplementary material for: Multiple Classes of Immune-Related Proteases Associated with the Cell Death Response in Pepper Plants
Source: PLoS One. 2013 May 16;8(5):e63533. doi: 10.1371/journal.pone.0063533 (PMC3656034; doi:10.1371/journal.pone.0063533)
Supplement: Table S2 — List of 153 protease-silenced plants with developmental phenotypes and responses to incompatible and compatible pathogen. a Developmental phenotypes abbreviations : N.D. = No difference, ISG = Inhibition of shoot growth, ISG+ALS = Inhibition of shoot growth with abnormal leaf shape, L = Lethality, LCC = Leaf color changed, ALS = Abnormal leaf shape. bResponse to incompatible pathogens abbreviations :+ = Delayed HR, − = Enhanced HR, N.D. = No difference, L = Lethality. cResponse to compatible pathogens abbreviations :+ = Delayed symptom, N.D. = No difference, L = Lethality. (DOCX) [file pone.0063533.s007.docx]

## Supporting Information Tables

**Table S2. List of 153 protease-silenced plants with developmental phenotypes and responses to incompatible and compatible pathogen.**

| **Pepper EST ID** | **Classification^a^** | **Developmental phenotypes^b^** | **Incompatible^c^** | **Compatible^d^** |
| --- | --- | --- | --- | --- |
| Ncn4200 | Cysteine | ISG | – | + |
| Ncn10993 | Cysteine | ISG + ALS | – | + |
| Ncn6185 | Cysteine | N.D. | – | + |
| Ncn7004 | Cysteine | ISG + ALS | + | + |
| Ncn1302 | Cysteine | ISG | N.D. | + |
| Ncn1555 | Cysteine | ISG | N.D. | + |
| Ncn8581 | Cysteine | ISG + ALS | N.D. | + |
| Ncn1007 | Metallo- | ISG + ALS | N.D. | + |
| Ncn8507 | Metallo- | ISG + ALS | N.D. | + |
| Ncn10583 | Metallo- | N.D. | N.D. | + |
| Ncn7594 | Serine | ISG | N.D. | + |
| Ncn3606 | Serine | N.D. | N.D. | + |
| Ncn3994 | Serine | N.D. | N.D. | + |
| Ncn665 | Serine | N.D. | N.D. | + |
| Ncn8285 | Serine | N.D. | N.D. | + |
| Ncn8849 | Threonine | ISG + ALS | N.D. | + |
| Ncn2134 | Threonine | L | L | L |
| Ncn2301 | Threonine | L | L | L |
| Ncn5127 | Threonine | L | L | L |
| Ncn5665 | Threonine | L | L | L |
| Ncn7753 | Threonine | L | L | L |
| Ncn806 | Threonine | L | L | L |
| Ncn2901 | Aspartic | ISG | – | N.D. |
| Ncn2258 | Aspartic | N.D. | + | N.D. |
| Ncn5964 | Aspartic | N.D. | + | N.D. |
| Ncn3506 | Cysteine | ISG | – | N.D. |
| Ncn5725 | Cysteine | ISG | – | N.D. |
| Ncn6060 | Cysteine | ISG | – | N.D. |
| Ncn6114 | Cysteine | ISG | – | N.D. |
| Ncn7910 | Cysteine | ISG | – | N.D. |
| Ncn1465 | Cysteine | N.D. | – | N.D. |
| Ncn2155 | Cysteine | N.D. | – | N.D. |
| Ncn9481 | Cysteine | N.D. | – | N.D. |
| Ncn1714 | Metallo- | ISG | – | N.D. |
| Ncn6721 | Metallo- | ISG | – | N.D. |
| Ncn13 | Metallo- | ISG + ALS | – | N.D. |
| Ncn5653 | Metallo- | ISG + ALS | – | N.D. |
| Ncn1877 | Metallo- | N.D. | – | N.D. |
| Ncn3988 | Metallo- | N.D. | – | N.D. |
| Ncn7241 | Metallo- | N.D. | – | N.D. |
| Ncn1998 | Serine | ISG | – | N.D. |
| Ncn10926 | Serine | N.D. | – | N.D. |
| Ncn4314 | Serine | N.D. | – | N.D. |
| Ncn8797 | Serine | N.D. | – | N.D. |
| Ncn9832 | Serine | N.D. | – | N.D. |
| Ncn8809 | Serine | ISG | + | N.D. |
| Ncn4597 | Serine | N.D. | + | N.D. |
| Ncn5036 | Serine | N.D. | + | N.D. |
| Ncn7446 | Serine | N.D. | + | N.D. |
| Ncn9855 | Serine | N.D. | + | N.D. |
| Ncn945 | Threonine | ISG | – | N.D. |
| Ncn1321 | Threonine | N.D. | – | N.D. |
| Ncn9390 | Aspartic | ALS | N.D. | N.D. |
| Ncn10694 | Aspartic | ISG | N.D. | N.D. |
| Ncn11220 | Aspartic | ISG | N.D. | N.D. |
| Ncn769 | Aspartic | ISG | N.D. | N.D. |
| Ncn7837 | Aspartic | ISG | N.D. | N.D. |
| Ncn8786 | Aspartic | ISG | N.D. | N.D. |
| Ncn9667 | Aspartic | ISG | N.D. | N.D. |
| Ncn9753 | Aspartic | ISG | N.D. | N.D. |
| Ncn1466 | Cysteine | ISG | N.D. | N.D. |
| Ncn2097 | Cysteine | ISG | N.D. | N.D. |
| Ncn7292 | Cysteine | ISG + ALS | N.D. | N.D. |
| Ncn4012 | Metallo- | LCC | N.D. | N.D. |
| Ncn4707 | Metallo- | LCC | N.D. | N.D. |
| Ncn6804 | Metallo- | LCC | N.D. | N.D. |
| Ncn7707 | Metallo- | ISG | N.D. | N.D. |
| Ncn7876 | Metallo- | ISG | N.D. | N.D. |
| Ncn8857 | Metallo- | ISG | N.D. | N.D. |
| Ncn918 | Metallo- | ISG + ALS | N.D. | N.D. |
| Ncn305 | Serine | ALS | N.D. | N.D. |
| Ncn3211 | Serine | LCC | N.D. | N.D. |
| Ncn3294 | Serine | LCC | N.D. | N.D. |
| Ncn390 | Serine | LCC | N.D. | N.D. |
| Ncn255 | Serine | ISG | N.D. | N.D. |
| Ncn2893 | Serine | ISG | N.D. | N.D. |
| Ncn3372 | Serine | ISG | N.D. | N.D. |
| Ncn4042 | Serine | ISG | N.D. | N.D. |
| Ncn4315 | Serine | ISG | N.D. | N.D. |
| Ncn5934 | Serine | ISG | N.D. | N.D. |
| Ncn650 | Serine | ISG | N.D. | N.D. |
| Ncn9653 | Serine | ISG | N.D. | N.D. |
| Ncn949 | Serine | ISG + ALS | N.D. | N.D. |
| Ncn7053 | Threonine | ALS | N.D. | N.D. |
| Ncn10366 | Metallo- | N.D. | N.D. | N.D. |
| Ncn10706 | Serine | N.D. | N.D. | N.D. |
| Ncn10708 | Serine | N.D. | N.D. | N.D. |
| Ncn10742 | Serine | N.D. | N.D. | N.D. |
| Ncn10829 | Serine | N.D. | N.D. | N.D. |
| Ncn1105 | Serine | N.D. | N.D. | N.D. |
| Ncn1106 | Serine | N.D. | N.D. | N.D. |
| Ncn11088 | Serine | N.D. | N.D. | N.D. |
| Ncn1175 | Cysteine | N.D. | N.D. | N.D. |
| Ncn1750 | Serine | N.D. | N.D. | N.D. |
| Ncn1929 | Serine | N.D. | N.D. | N.D. |
| Ncn1954 | Serine | N.D. | N.D. | N.D. |
| Ncn1999 | Serine | N.D. | N.D. | N.D. |
| Ncn2083 | Cysteine | N.D. | N.D. | N.D. |
| Ncn2099 | Cysteine | N.D. | N.D. | N.D. |
| Ncn2110 | Aspartic | N.D. | N.D. | N.D. |
| Ncn2132 | Metallo- | N.D. | N.D. | N.D. |
| Ncn2327 | Serine | N.D. | N.D. | N.D. |
| Ncn2393 | Cysteine | N.D. | N.D. | N.D. |
| Ncn2523 | Metallo- | N.D. | N.D. | N.D. |
| Ncn259 | Serine | N.D. | N.D. | N.D. |
| Ncn2967 | Metallo- | N.D. | N.D. | N.D. |
| Ncn2971 | Serine | N.D. | N.D. | N.D. |
| Ncn304 | Serine | N.D. | N.D. | N.D. |
| Ncn3373 | Serine | N.D. | N.D. | N.D. |
| Ncn3963 | Serine | N.D. | N.D. | N.D. |
| Ncn3992 | Serine | N.D. | N.D. | N.D. |
| Ncn4099 | Serine | N.D. | N.D. | N.D. |
| Ncn4141 | Metallo- | N.D. | N.D. | N.D. |
| Ncn4199 | Cysteine | N.D. | N.D. | N.D. |
| Ncn4906 | Cysteine | N.D. | N.D. | N.D. |
| Ncn5180 | Cysteine | N.D. | N.D. | N.D. |
| Ncn5328 | Serine | N.D. | N.D. | N.D. |
| Ncn5885 | Serine | N.D. | N.D. | N.D. |
| Ncn6037 | Serine | N.D. | N.D. | N.D. |
| Ncn6073 | Serine | N.D. | N.D. | N.D. |
| Ncn6260 | Serine | N.D. | N.D. | N.D. |
| Ncn642 | Serine | N.D. | N.D. | N.D. |
| Ncn651 | Serine | N.D. | N.D. | N.D. |
| Ncn652 | Serine | N.D. | N.D. | N.D. |
| Ncn6706 | Serine | N.D. | N.D. | N.D. |
| Ncn6818 | Serine | N.D. | N.D. | N.D. |
| Ncn6835 | Metallo- | N.D. | N.D. | N.D. |
| Ncn707 | Serine | N.D. | N.D. | N.D. |
| Ncn708 | Serine | N.D. | N.D. | N.D. |
| Ncn7277 | Serine | N.D. | N.D. | N.D. |
| Ncn7288 | Threonine | N.D. | N.D. | N.D. |
| Ncn7295 | Metallo- | N.D. | N.D. | N.D. |
| Ncn7368 | Serine | N.D. | N.D. | N.D. |
| Ncn7420 | Aspartic | N.D. | N.D. | N.D. |
| Ncn75 | Cysteine | N.D. | N.D. | N.D. |
| Ncn7704 | Serine | N.D. | N.D. | N.D. |
| Ncn7737 | Serine | N.D. | N.D. | N.D. |
| Ncn8210 | Serine | N.D. | N.D. | N.D. |
| Ncn8326 | Metallo- | N.D. | N.D. | N.D. |
| Ncn8350 | Serine | N.D. | N.D. | N.D. |
| Ncn8392 | Serine | N.D. | N.D. | N.D. |
| Ncn8762 | Cysteine | N.D. | N.D. | N.D. |
| Ncn881 | Metallo- | N.D. | N.D. | N.D. |
| Ncn8815 | Serine | N.D. | N.D. | N.D. |
| Ncn8884 | Aspartic | N.D. | N.D. | N.D. |
| Ncn8903 | Metallo- | N.D. | N.D. | N.D. |
| Ncn9014 | Serine | N.D. | N.D. | N.D. |
| Ncn9260 | Serine | N.D. | N.D. | N.D. |
| Ncn964 | Serine | N.D. | N.D. | N.D. |
| Ncn9826 | Metallo- | N.D. | N.D. | N.D. |
| Ncn9846 | Aspartic | N.D. | N.D. | N.D. |
| Ncn9856 | Serine | N.D. | N.D. | N.D. |
| Ncn9857 | Serine | N.D. | N.D. | N.D. |

^a^ Developmental phenotypes abbreviations : N.D. = No difference, ISG = Inhibition of shoot growth, ISG + ALS = Inhibition of shoot growth with abnormal leaf shape, L = Lethality, LCC = Leaf color changed, ALS = Abnormal leaf shape

^b^ Response to incompatible pathogens abbreviations : + = Delayed HR, - = Enhanced HR, N.D. = No difference, L = Lethality

^c^ Response to compatible pathogens abbreviations : + = Delayed symptom, N.D. = No difference, L = Lethality
