# Supplementary material for: Multiple Classes of Immune-Related Proteases Associated with the Cell Death Response in Pepper Plants
Source: PLoS One. 2013 May 16;8(5):e63533. doi: 10.1371/journal.pone.0063533 (PMC3656034; doi:10.1371/journal.pone.0063533)
Supplement: Table S3 — Primer information for quantitative RT-PCR analysis. (DOCX) [file pone.0063533.s008.docx]

## Supporting Information Tables

**Table S3. Primer information for quantitative RT-PCR analysis**

| **Pepper EST ID** | **Sequence information (5' -> 3')** | | **Reference** |
| --- | --- | --- | --- |
|  | **Forward** | **Reverse** |  |
| ***NbActin*** | **CCAGGTATTGCTGATAGAATGAG** | **CTGAGGGAAGCCAAGATAGAG** | **New Phytol. 2009;184(1):71-84** |
| **Ncn2132** | **GCAACTCCTCTCGATTAGGG** | **ACGAGCGCATGAAGTTAGGT** |  |
| **Ncn881** | **GGGTAGTGAAGTGGCTCCAA** | **CTTTCACAGCCCCACAAGAT** |  |
| **Ncn6721** | **GCTTCCGTTGTTTCTGAAGG** | **TTCTTCTTTGCGGCTTCTGT** |  |
| **Ncn9826** | **ATGGTGTTGGAGCTGCACTT** | **TCCAAAACGATTCGGTGTATC** |  |
| **Ncn5036** | **CCTGTTGCCAAGCTGAAGAT** | **GGAATGTGTGTGCTCCTTTTG** |  |
| **Ncn964** | **CATGAAAGGGTCAACGATTTG** | **GCTGAAAGGGTCAATTTTCG** |  |
| **Ncn8326** | **CGCCAAGGTTGGTTCAGA** | **TAGGGGTGAACTCCATGAGC** |  |
| **Ncn10583** | **CACGAGGGCACAGCATAAGA** | **CCCATCAAGCTGTGAGAACT** |  |
